# Synthesis and Self-Assembling Properties of Carbohydrate- and Diarylethene-Based Photoswitchable Molecular Gelators

**DOI:** 10.3390/molecules28176228

**Published:** 2023-08-24

**Authors:** Pramod Aryal, Joedian Morris, Surya B. Adhikari, Jonathan Bietsch, Guijun Wang

**Affiliations:** Department of Chemistry and Biochemistry, Old Dominion University, 4501 Elkhorn Avenue, Norfolk, VA 23529-0126, USA; paryal@odu.edu (P.A.); jymorris@odu.edu (J.M.); sbadhika@odu.edu (S.B.A.); jbietsch@odu.edu (J.B.)

**Keywords:** glycolipids, gelators, carbohydrate, dye absorption, diarylethene, photochromic compounds, molecular gelators, photoswitchable gels

## Abstract

Carbohydrate-based low-molecular-weight gelators are interesting new materials with many potential applications. These compounds can be designed to include multiple stimuli-responsive functional groups. In this study, we designed and synthesized several chemically responsive bola-glycolipids and dimeric carbohydrate- and diarylethene-based photoswitchable derivatives. The dimeric glycolipids formed stable gels in a variety of solvent systems. The best performing gelators in this series contained decanedioic and dithienylethene (DTE) spacers, which formed gels in eight and nine of the tested solvents, respectively. The two new DTE-containing esters possessed interesting photoswitching properties and DTE derivative **7** was found to have versatile gelation properties in many solvents, including DMSO solutions at low concentrations. The gels formed by these compounds were stable under acidic conditions and tended to hydrolyze under basic conditions. Several gels were used to absorb rhodamine B and Toluidine blue from aqueous solutions. In this study, we demonstrated the rational design of molecular gelators which incorporated photoresponsive and pH responsive functions, leading to the discovery of multiple effective stimuli-responsive gelators.

## 1. Introduction

Stimuli-responsive molecular assemblies have gained much recognition as useful novel functional materials. Among the many different types of molecular assemblies, low-molecular-weight gelators (LMWGs) are a fascinating class of compounds which can be used to form supramolecular gels with different properties and applications. The formation of the resulting supramolecular gels from LMWGs relies on non-covalent intermolecular forces, including hydrogen bonding, hydrophobic interactions, π–π stacking, and van der Waals interactions, etc. [1]. The gels formed by LMWGs have been explored as new materials with applications in various fields such as biomedical applications [2], drug delivery systems [3], optical and electronic materials [4], and the removal of pollutants [5]. Molecular gelators obtained from natural sources, such as carbohydrates, are especially useful new materials. Carbohydrates have been explored extensively to form supramolecular gels. The intrinsic chirality and biocompatibility of sugar-derived self-assembling systems lend them to many practical applications, including environmental applications and controlled-release delivery systems [6,7]. The conversion of sugars into useful functional materials using simple modifications to their original structure is a valuable strategy for the development of new advanced materials. 

**Figure 1:**
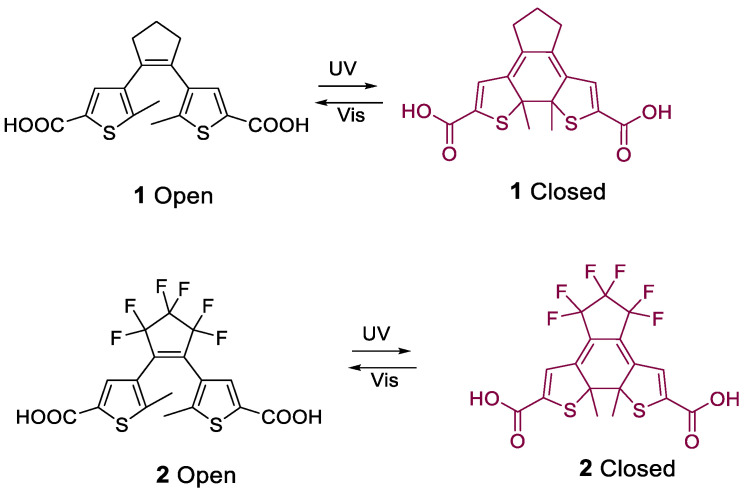
Structure of dithienylethene carboxylic acids.

The formation of supramolecular gels containing designed structures that are responsive to various stimuli is a well-studied method for creating novel functional materials. The typical gels formed by LMWGs are reversible and temperature sensitive, as they are in a solution phase at elevated temperatures and self-assemble to form gels at cooler temperatures. Many functionalities have been incorporated in the structure-based design of new molecular gelators, including functional groups sensitive to pH, light, and chemical stimuli. Among these various stimuli-responsive LMWGs, photoresponsive gelators are exceptionally interesting new materials showing great promise for practical applications. Utilizing light as a trigger can induce new material properties and has the potential for practical applications in interesting interdisciplinary applications. There are many photoresponsive functional groups that have been utilized in the design of molecular gelators including but not limited to azobenzene, diacetylene, and diarylethene [8,9,10,11]. Diarylethene (DAE)- or dithienylethene (DTE)-based molecular switches have gained great attention in recent years [12,13,14,15,16,17]. DAE-based systems have unique advantages and properties because they can form a stable cyclized isomer upon photo irradiation. Both the open and closed isomers are stable and can be interconverted with different stimuli [18]. Figure 1 depicts the isomerization of dithienylethene-based dicarboxylic acid **1** and the hexafluorinated derivative **2**. Upon treatment with UV light, the DTE diacids are converted to the closed isomers that have extended conjugation, which produces red or blue colored products. 

Diarylethene (DAE) or dithienylethene (DTE) photochromic functions have been integrated in the design of stimuli responsive supramolecular gels. Two main types of photoresponsive DAE-based supramolecular gels have been studied. The first class incorporates the DAE molecules with a known gelator to form a two-component gel system [19,20,21]. The DAE photoswitches are used to modulate the properties of the resulting gels. Bispyridinium dithienylethene derivatives have been applied in the formation of various hybrid gels with polymers or metal ions and other ligands to form photochromic supramolecular gels [22,23,24,25,26]. In the second main class of DAE-based gelators, the diarylethene functionality is part of the gelator structure, where the diarylethene moieties have been functionalized with a variety of other functional groups to form a low-molecular-weight gelator. Feringa et al. have reported several different DAE-based photoswitchable organogelators [27,28,29,30]. Tian et al. also reported DTE-based organogelators which contain a fluorescent naphthylamide functional group [31] or metal ion recognition unit [32]. Besides these studies, several other dithienylcyclopentene-based low-molecular-weight organogelators have been synthesized and reported [33,34]. There are not many DTE-based low-molecular-weight hydrogelators due to the hydrophobic properties of the DTE system. A series of maleimide-based dithienyl switches incorporating tripeptides was reported to form hydrogels at 20–40 mg/mL [35]. Recently, Feringa et al. designed single-stranded DNA-based hydrogels which contain a diarylethene photoswitch [36]. Sodium salts of 1,2-bisthienylperfluorocyclopentene have also been covalently functionalized to form gels in DMF and DMSO [37]. Recently, an interesting photochromic LMWG which contains a hexafluorocyclopentene diethienyl moiety (F6DTE) with a covalently linked terpyridine moiety was studied, and the gelator formed assemblies with lanthanide ions that formed coordination polymeric gels [38]. 

**Figure 2:**
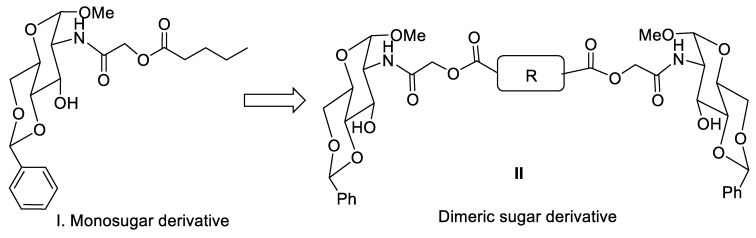
Structures of sugar-based LMWG **I** and the dimeric derivatives **II**.

Despite the examples of DTE-based LMWGs, to the best of our knowledge, using a sugar template to form a new DTE-based gelator has not been reported yet. Sugar-based DTE glycoconjugates have been designed and utilized for a variety of biomedical applications [39,40]. We are intrigued by the possibility of creating a novel class of photoswitchable carbohydrate-based gelators by incorporating DAE units into the sugar gelator template. As shown in Figure 2, we have previously prepared monomeric glucosamide esters (I) that were excellent gelators for many solvents, and these gelators showed potential applications in the biomedical and environmental sciences [41]. In this research, in order to design carbohydrate-based gelators with enhanced molecular assembling properties, we envisioned that dimeric derivatives (II) may increase intermolecular interactions and, therefore, result in more stable gels. Moreover, the differences in the hydrophobicity of the linkers between the sugar headgroups may also affect the gelation properties. Incorporating a carbohydrate moiety and the diarylethene photoswitching functions into a new molecular design can lead to interesting new materials. 

## 2. Results and Discussions

**Scheme 1:**
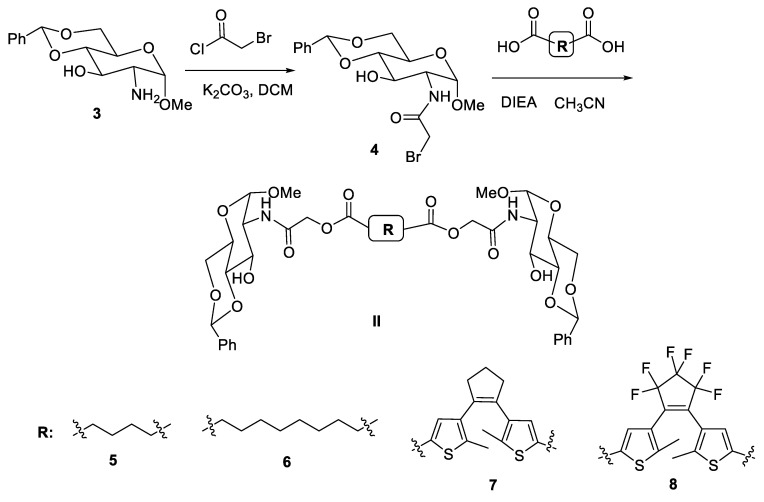
Synthesis of carbohydrate-based ester derivatives **5–8**.

The synthesis of the dimeric glycolipids is shown in Scheme 1. Glucosamine derivatives **3** and **4** were prepared following a procedure in the literature [1]. The dimeric glycolipids **II** were synthesized using nucleophilic substitutions of the bromo group by carboxylate groups. Four derivatives were synthesized, including the linear alkyl spacer derivatives **5** and **6**, and incorporating photochromic acids **1** and **2** resulted in dimers **7** and **8** in good yields. 

After these compounds were synthesized and purified, their gelation properties were evaluated in a series of solvents; the results are included in Table 1. The linear alkyl chain spacer derivatives **5** and **6** were first synthesized and evaluated for their gelation properties. The short-chain analog **5** formed gels in five different solvents. Increasing the methylene spacer from four to eight led to compound **6**, which showed excellent gelation properties at relatively low concentrations, especially in alcohols and ethanol water mixtures or DMSO water (*v*/*v* 1:2). Diesters **7** and **8** contained covalently linked diacids **1** and **2**. Interestingly, like compound **6**, compound **7** was a versatile gelator for nine different solvents. The dithienylethene (DTE)-containing diester was especially effective for DMSO water mixtures, forming gels at 1.6 mg/mL concentrations. In contrast, the F6-DTE derivative **8** tended to be more soluble in organic solvents and only formed gels in several alcohols at higher concentrations. The gels mostly appeared opaque to translucent.

**Figure 3:**
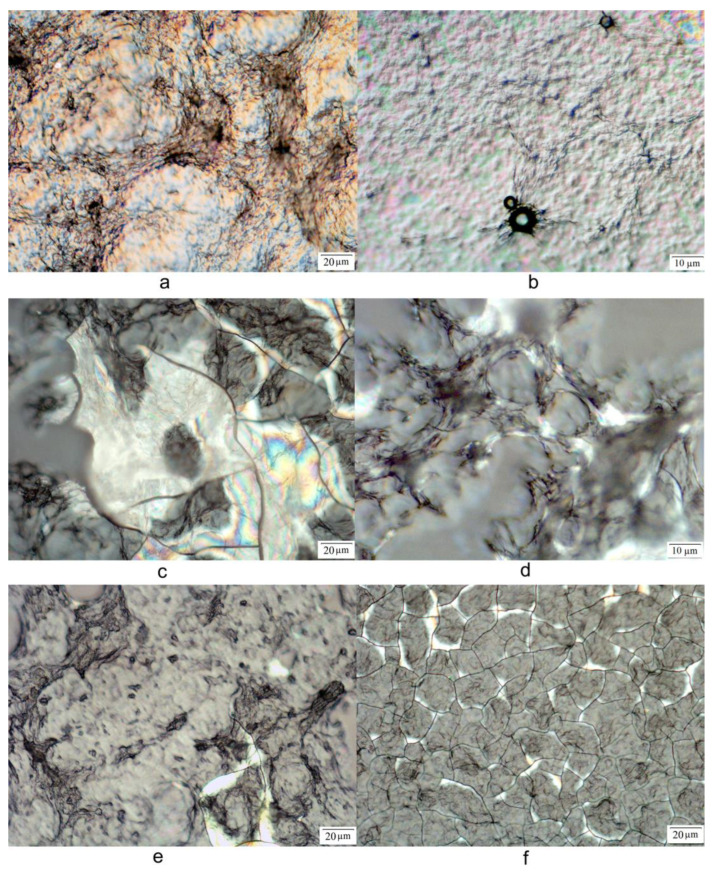
Optical microscope images of several gels. (**a**) Compound **5** in DMSO/H_2_O (*v*/*v* 1:2) at 5.0 mg/mL, (**b**) compound **7** in DMSO/H_2_O (*v*/*v* 1:2) at 1.6 mg/mL, (**c**) compound **7** in isopropanol at 6.6 mg/mL, (**d**) compound **7** in n-butanol at 6.6 mg/mL, (**e**) compound **8** in ethanol at 13.3 mg/mL, (**f**) compound **8** in *n*-butanol at 20 mg/mL.

The optical microscopy images of several gels are included in Figure 3. Interestingly, these bipolar glycolipids exhibited different types of morphology in comparison to the majority of mono-glucosamine-based LMWGs, which typically formed more distinct fibers. The gels formed by new bola-glycolipids mostly appeared as smooth films, amorphous gel pieces or ribbons. The gel of compound **5** in DMSO/H_2_O (*v*/*v* 1:2) appeared as an uneven film, with certain areas having bundled ribbons (Figure 3a). Compound **7** in DMSO/H_2_O (*v*/*v* 1:2) showed a similar smooth film-like morphology, but large circular shapes were observed in certain areas (Figure 3b). Compound **7** in isopropanol and n-butanol appeared to have similar morphologies, with more distinct long ribbons and circular fibers bundled together (Figure 3c,d). The hexafluorinated derivative **8** formed gels in ethanol and n-butanol at higher concentrations. The gel in ethanol exhibited similar morphologies to those of the gels formed by compound **7** and also exhibited smooth films with long wires in certain regions (Figure 3e). The gel in n-butanol exhibited mostly crosslinked domains of large ribbons and smaller fibers inside the cross-linked wires (Figure 3f).

**Figure 4:**
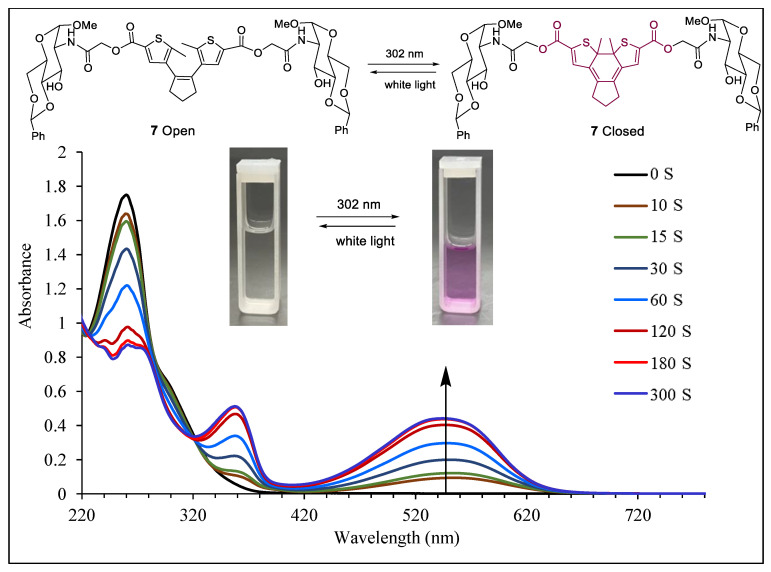
The UV-Vis spectra of compound **7** under UV irradiation at different time points. λ = 260 nm for **7O**, and 358 and 545 nm for closed form **7C**, with the isosbestic point at 321 nm.

**Figure 5:**
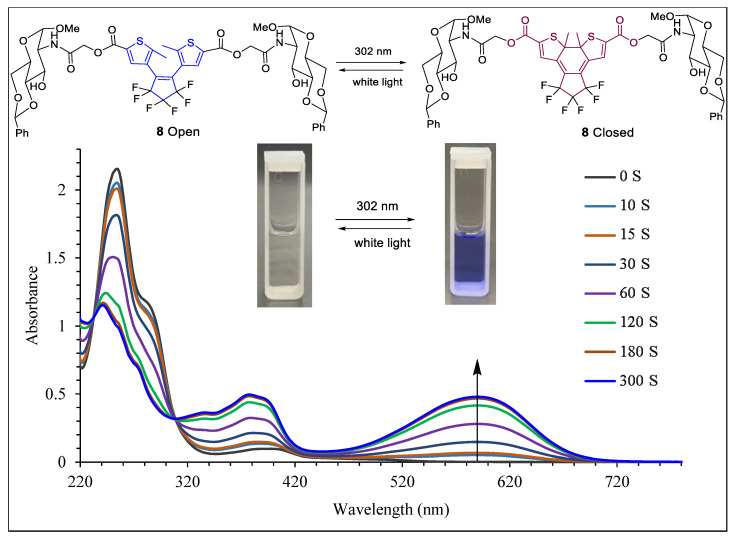
The UV-Vis spectra of compound **8** under UV irradiation at different time points. λ = 378, 595 nm for the closed form; 252 and 280 nm for the open form; isosbestic points at 232 and 308 nm.

The DTE-containing compounds **7** and **8** were also evaluated for their photochromic properties. The UV-Vis spectra of compounds **7** and **8** before and after irradiation are shown in Figure 4 and Figure 5. By default, we refer to compounds **7** and **8** as their open forms, which can also be denoted as **7O** and **8O**, respectively. The closed or cyclized forms are denoted as **7C** and **8C**. The open forms of both compounds were colorless, while after irradiation with 302 nm UV light compound **7** appeared pink-to-purple in color (Figure 4). The absorption at 260 nm for **7O** decreased and two new signals at 358 and 545 nm increased for the closed form **7C**; the isosbestic point at 321 nm was observed. After approximately 3 min of irradiation, signal intensities reached their highest point. 

After irradiation with 302 nm UV light, compound **8** appeared dark blue in color. Compound **8O** had two absorption wavelengths of 252 and 280 nm; after irradiation, these peaks decreased and the new peaks for the closed form **8C** appeared at 378 and 595 nm, while the isosbestic points at 232 and 308 nm were observed. At approximately 5 min of irradiation, the signal intensities reached their maximum. 

**Figure 6:**
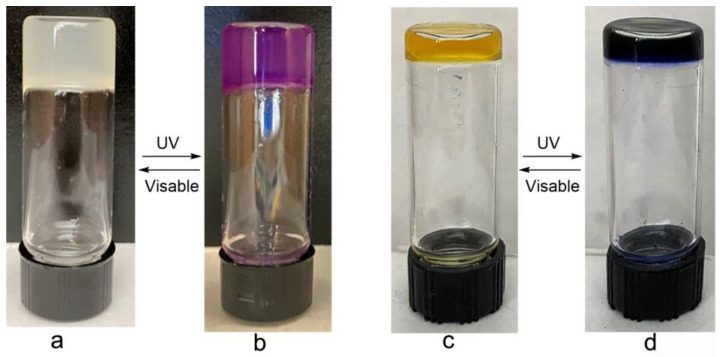
Gel photographs before and after UV irradiation: (**a**) An opaque gel formed by compound **7** in DMSO:H_2_O (*v*/*v* 1:2) at 3.3 mg/mL. (**b**) The gel of **7** in DMSO:H_2_O (*v*/*v* 1:2) after UV light at 302 nm for 15 min. (**c**) A transparent gel formed by compound **8** in ethanol at 13.3 mg/mL. (**d**) The gel formed by **8** in ethanol after irradiation with UV light at 302 nm for 15 min.

Not only did the solutions of these two compounds exhibit reversible photochromism, the gels formed by them were also tested for photoswitching properties. After UV irradiation, the gels exhibited isomerization to the cyclized form or the closed form. A few examples are shown in Figure 6. The gel of compound **7** appeared opaque and after UV treatment it turned deep purple (Figure 6b). The gel formed by compound **8** in ethanol appeared orange under visible light (Figure 6c), and it turned dark blue upon UV treatment (Figure 6d).

**Scheme 2:**
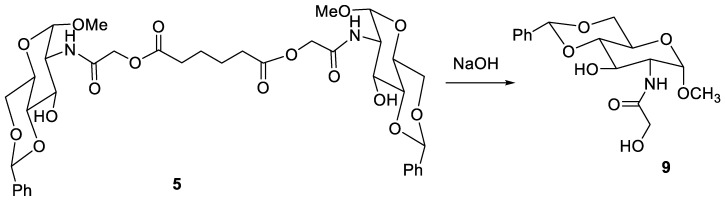
Cleavage of the dimeric esters in the presence of a base.

As the efficient gelators contained ester linkages, we expected that the gels could be stimuli responsive through hydrolysis of the ester bond under basic conditions. Gelator **5** had a relatively simple structure and was used for a gel stability study under basic conditions. Scheme 2 illustrates the cleavage of the respective short chain ester dimers under basic conditions to give the hydrolyzed product **9**, whose gel test results were previously reported [41]. The gels of compound **5** were made in DMSO:H_2_O (*v*/*v*, 1:1) at a concentration of 8.0 mg/mL, after which they were allowed to sit for 2 h before adding the different pH solutions. At mild basic conditions of pH 12, full hydrolysis was seen for the gel of compound **5** after 120 h. The gel photos are included in ESI Figure S9. At pH 12, the gel appeared to disintegrate partially after 4 h, and did not fully dissolve until reaching 120 h, at which time the gelator was extracted and analyzed using ^1^H NMR spectroscopy. The NMR spectrum of the extracted crude compound is shown in Figure S10, which corresponds to the hydrolyzed product **9**. This confirmed that the diesters could be hydrolyzed under basic conditions, and that if the compound forms a gel then it could be base-triggered to form a solution.

Gelator **5** was also tested for its stability under acidic conditions, since the compound has an acetal functional group. Interestingly, under acidic conditions, the gels were still quite stable after 18 days. The recordings of their appearances are included in Table S1 and selected gel photos are included in ESI Figures S11 and S12 for pH 2 and pH 4, respectively. These results revealed that the gels were stable under neutral and mild acidic conditions; however, they could be chemically converted to solutions in the presence of bases, and this can be further studied for application in stimuli responsive dimeric esters. 

## 3. Dye Absorption Studies

**Figure 7:**
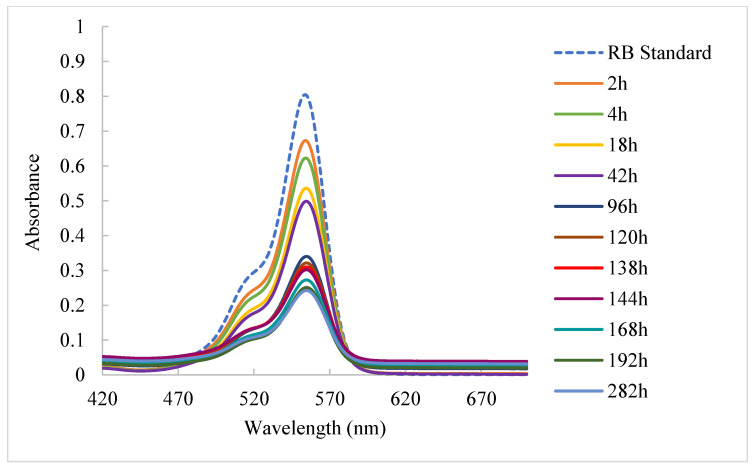
Time-dependent UV-Vis spectra of the rhodamine B base solution above the gel of compound **6**.

To evaluate the possible utilities of these dimeric gelators in the removal of toxic dyes, two representative gelators were selected for dye removal/absorption experiment. Compounds **6** and **7** were selected for the experiment. A 2 mL gel formed by compound **6** in DMSO:H_2_O (*v*/*v* 1:2) was prepared first and was left standing for sometime between 30 min and 1 h; then, 2 mL of the aqueous rhodamine B base solution 0.0082 mM was placed on top of the gel. The dye remaining in the aqueous phase was monitored over time via UV-Vis spectroscopy. The time-dependent absorption spectra using gelator **6** are depicted in Figure 7. The gel photos and absorbance profiles of the dye using the gel are included in ESI Figures S13–S15. The results demonstrated that these gels could absorb toxic dyes from aqueous solutions. The gel of compound **6** had an absorption efficiency of approximately 70% after 282 h. 

**Figure 8:**
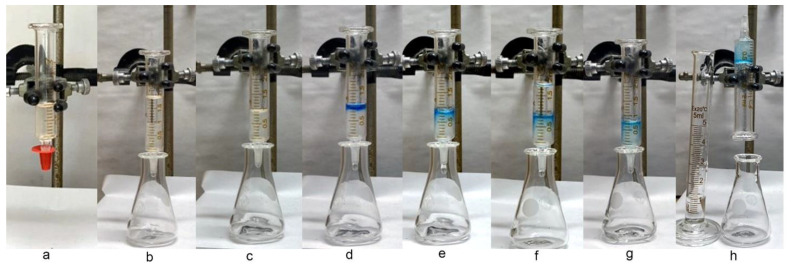
Dye absorption study using compound **7** open. (**a**) A gel column formed by compound **7** in 1.0 mL DMSO:H_2_O (*v*/*v* 1:2) at 4.0 mg/mL; (**b**) 1 mL DI water was added to the gel column; (**c**) the gel column after water was eluted; (**d**) the gel column with 0.1 mL Toluidine blue dye solution (50 mg/L) added; (**e**) after the dye solution was eluted; (**f**) 1.0 mL water was added to the gel column in (**e**,**g**) after water flush; (**h**), inverted gel column in (**g**).

**Figure 9:**
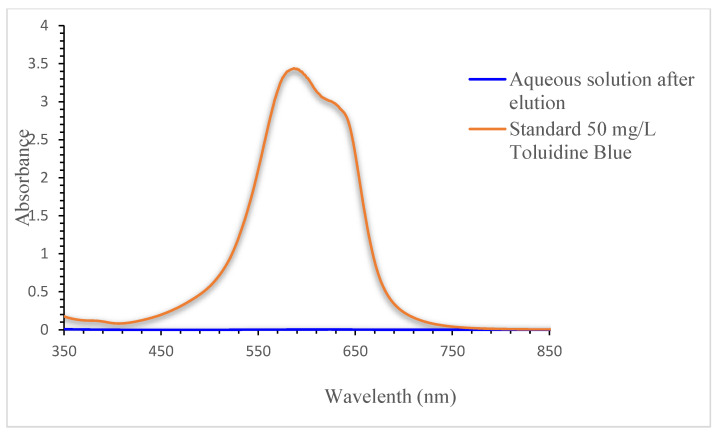
The UV-Vis spectra of the 50.0 mg/L TBO solution loaded to top of the gel by compound **7** in Figure 8d and the collected aqueous solution after elution in Figure 8h.

The gels formed by compound **7** were also evaluated for dye removal applications. Two different dyes, Toluidine blue and rhodamine B, were tested. The results are shown in Figure 8 and Figure 9 and ESI Figures S16–S23. For these experiments, gels were prepared in a syringe column and the dye solutions were “filtered” using the gel column. For the removal of Toluidine blue, both the open and closed forms of compound **7** were employed, whereas only the open form was used for rhodamine B. As shown in Figure 8, the gel formed by compound **7** was prepared inside a syringe column. The gel column was then used to elute the Toluidine blue dye solutions. The gel column was stable and could be inverted at the end of the experiment (Figure 8a–h). The UV-Vis spectra of the collected aqueous phase after elution using compound **7O** and **7C** are shown in Figure 9 and Figure S20, respectively. The estimated amount of Toluidine blue dye removed by the gel column was 95% for the open form and 96% for the closed form, which indicated that both the open and cyclized forms of the gelators were effective at absorbing the dye molecules. 

The gel column prepared by compound **7** was also used to remove rhodamine B from the aqueous solution using the same method. The results are included in Figures S22 and S23. The gel column was able to remove approximately 99.5% of the rhodamine B from its aqueous solution. Therefore, the gels demonstrated effectiveness at removing certain water-soluble dyes from solutions effectively. 

## 4. Conclusions

We have designed and synthesized a series of four dimeric glycolipids that are functional low-molecular-weight gelators. All compounds exhibited gelation properties in at least one of the selected solvents. These gelators are responsive to base triggers, the gels formed by compound **5** turned into solutions after treating with mild bases. Compounds **6** and **7** were the most versatile gelators, forming gels in eight and nine different solvents, respectively. They showed gelation properties in alcohols, ethanol water mixtures, and DMSO water mixtures. More importantly, two novel diarylethene-based derivatives were also prepared. The DTE derivative compound **7** was an efficient gelator forming gels in many solvents. In contrast, the fluorinated derivative **8** showed diminished gelation tendencies. Both compounds exhibited reversible photochromism upon UV light and visible light irradiation. Interestingly, the resulting gels also showed reversible photochromism and both open and the closed forms maintained the gel state. The gel formed by compound **6** was effective at absorbing rhodamine B dye solution. The DTE gelator **7** was also used for dye removal studies, and the gels formed by both open and closed forms showed that they could absorb over 95% of the Toluidine blue or rhodamine B from aqueous mixtures. These novel carbohydrate-based gelators are expected to have other potential applications besides the dye absorption. Our study has demonstrated the creation of a new class of multiple stimuli-responsive LMWGs. 

## 5. Experimental Section

### 5.1. General Method and Materials

Reagents and solvents were used as they were received from the suppliers. All purifications were conducted using flash column chromatography with 230–400 mesh silica gel obtained from Natland International Corporation, unless otherwise noted. The deacetylation reaction was performed in a Mars 6 microwave reactor from CEM Corporation. Nuclear magnetic resonance (NMR) analysis was conducted using a 400 MHz Bruker NMR spectrometer. Melting point measurements were carried out using a Fisher–Johns Melting Point apparatus. UV-Vis experiments were performed using a SHIMADZU UV-1800 Spectrophotometer. The LCMS data were obtained using an Agilent 6120B Single Quad mass spectrometer and a LC1260 HPLC system. High-resolution mass spectra (HRMS) were obtained with a Thermo Scientific LTQ Discovery spectrometer using +ESI and reported for the molecular ion [M + Na]^+^.

Optical Microscopy: A small amount of gel was transferred onto a clean glass slide and then left to air dry for approximately a day. The gel was then observed under an Olympus BX60M optical microscope at brightfield using an Olympus DP73-1-51 high-performance 17 MP digital camera with pixel shifting and Peltier cooling. The program used to acquire and store the images was CellSens Dimension 1.11.

Gelation test: Approximately 2 mg of the desired compound was placed in a one-dram vial and 0.1 mL of the gelation solvent or solution was placed inside the vial to attain a concentration of 20 mg/mL. The vial was then heated until the gelator dissolved fully; sometimes the mixture was sonicated if necessary to help with dilution. After dissolving, it was left to cool for approximately 15 min or longer for the gel to form. After this period, if the sample was clear, it was recorded as soluble; if solid reappeared, it was recorded as a precipitate; if the sample formed a gel, then the vial was inverted; if there was no solvent flowing this indicated a stable gel; otherwise, it was recorded as an unstable gel. If gelation occurred, another 0.1 mL was added, and the method was repeated until an unstable gel formed. The MGC, which is the concentration prior to unstable gelation, was obtained.

Photoswitching experiment: The photoswitching study was carried out using a 6 W UVM-16 EL series UV Lamp with 302 nm and white light. For the solution experiment, typically 1 mmol solution of compounds **7** or **8** was dissolved in 1 mL acetonitrile, and this was diluted to 0.1 M. Then, 2.0 mL of the 0.1 M solution was added to a cuvette for UV-Vis measurement. The photo irradiation was performed using 302 nm UV light on top of the cuvette at different time points. The closed form was converted to the open form by treating it with white light for approximately 20 min. 

Base and Acid Stability Observations: A total of 4 mg of compound **5** was weighed out in a one-dram vial and 0.5 mL of DMSO:H_2_O (*v*/*v*, 1:1) was added to make the gel (two gels were made). After 2 h, 0.5 mL of different pH solutions was added to the vials and the gels were observed every hour to record any decomposition that occurred. If the gel became unstable, the resulting aqueous mixture was extracted with 2 mL of DCM to obtain a proton NMR spectrum. 

### 5.2. Dye Absorption Studies

#### 5.2.1. For Gelator **6** in a Bi-Phase System for Rhodamine B

A 0.004 mM solution of rhodamine B base solution was made by diluting a stock solution (0.0082 mM) by adding 4 mL of the stock solution to 18 mL of DI water to make a total volume of 20 mL. The stock solution was made by dissolving 0.0018 g of the rhodamine B base compound and dissolving it in 100 mL of DI water. From the diluted solution, 2 mL was placed on top of the gel for the study. The gels were prepared using 10 mg of compound **6** in 2.0 mL of DMSO:H_2_O (*v*/*v* 1:2). They were allowed to sit for 30 min to an hour before the start of the experiment. The absorbance was taken at different time intervals for the aqueous dye solutions on top of the gels.

#### 5.2.2. For Gelator **7** in a Gel Column

The gels formed by compound **7** in 4 mg/mL DMSO:H_2_O 1:2 were evaluated for their absorption of Rhodamine B dye. Gel was flushed with 1 mL water to swap the DMSO present in the gel. RB 0.1 mL (20 mg/L) was added to the gel and let settle for half an hour then when the dye was finished passing followed by adding 1 mL water and eluted 1.2 mL liquid was collected. The gel was stable enough to be inverted at the end of the experiment. Absorbance at λ_max_ (554 nm) of Rhodamine B was measured using a calibration curve. 

#### 5.2.3. Toluidine Blue Dye Absorption Studies

The gels formed by compound **7** in 4 mg/mL DMSO:H_2_O 1:2 were evaluated for their absorption of Toluidine blue dye. The solution was heated and then after cooling transferred into the gel column and left to settle for 14 h. For the closed form study, the solution was irradiated under UV 302 for 30 min to close the ring and heated. Then, after cooling it was transferred into the gel column and left to settle for 14 h in a dark room. In both the open and closed form column, 1 mL DI water was added on top of the column to flush DMSO from the gel column. Then, Toluidine blue dye 0.1 mL (50 mg/L) was added to 1 mL gel and left to settle for 30 min. When the dye finished passing, 1 mL water and 1.2 mL eluted liquid was added and diluted to 2.2 mL. The gel was stable enough to be inverted at the end of the experiment. Absorbance at λ_max_ (630 nm) of Toluidine blue was measured using a calibration curve.

### 5.3. Synthesis of Compounds ***5***–***8***

General procedure for the derivative **5–8** synthesis from headgroup **4**

Compound **4** was synthesized using a previously reported procedure [41]. In a 50 mL round bottom flask equipped with a drying tube the carboxylic acid (1.0 equivalents) was added to the flask, followed by diisopropylethylamine (DIEA) (2.0 equivalents) and 1–2 mL of anhydrous acetonitrile/DMF. The reaction mixture was stirred at room temperature for 30 min and then the headgroup, compound **4** (approximately 100 mg, 0.24 mmol, 2 equivalents), was added with 1–2 mL of anhydrous acetonitrile/DMF. The reaction mixture was then stirred for approximately 6–24 h at 70–75 °C, at which time TLC and ^1^H NMR spectroscopy were used to monitor the progress of the reaction. If not complete, the reaction mixture was stirred for longer. After the starting material was fully converted to the product, the reaction mixture was cooled and concentrated on a rotavap to remove the solvent. The crude product was worked up with DCM and 5% NaHCO_3_ solution, followed by water. The organic phase was dried over anhydrous sodium sulfate and the solvent was removed to obtain the crude product. The crude product was purified using flash column chromatography on silica gel using a gradient of dichloromethane and methanol. The quantities of reagents and characterization data are given below, but detailed procedures are not given unless different conditions were used. 

Synthesis of compound **5.** Adipic acid (0.0134 g, 0.092 mmol, 1 equiv.) followed by DIEA (0.069 mL, 0.41 mmol, 2 equiv.) and 2 mL of anhydrous acetonitrile were added in a 50 mL round bottom flask and the reaction mixture was allowed to stir at room temperature for 25 min. After this, compound **4** (0.0815 g, 0.20 mmol, 2.2 equiv.) was added along with 4.5 mL of anhydrous acetonitrile. The reaction mixture was left to stir for 25 h at 75 °C. Then, the solvent was removed from the reaction mixture via the rotavap. Workup was performed using DCM (15 mL × 3)/saturated NaHCO_3_ (10 mL), and then water. The combined organic layer was dried over Na_2_SO_4_ (anhydrous), filtered, and concentrated to give the crude product, which was purified using column chromatography using eluent from pure DCM to 3.5% MeOH/DCM to afford a white solid (67.1 mg, 93%) as the desired product. (R_f_ = 0.4 in 5% MeOH/DCM). mp 209.0–211.0 °C. ^1^H NMR (400 MHz, CDCl_3_) *δ* 7.54–7.46 (m, 4H, Ph-H), 7.42–7.32 (m, 6H, Ph-H), 6.37 (d, *J* = 8.5 Hz, 2H, NH), 5.52 (s, 2H, Ph-CH-), 4.74 (d, *J* = 3.8 Hz, 2H, H-1), 4.66–4.51 (m, 4H, -CH_2_-O), 4.32–4.16 (m, 4H, H-6a, H-2), 3.90 (t, *J* = 9.5 Hz, 2H, H-3), 3.83–3.69 (m, 4H, H-5, H-6b), 3.50 (dd~t, *J* = 8.9 Hz, 2H, H-4), 3.40 (s, 6H, OCH_3_), 3.07 (bs, 2H, 3-OH), 2.56–2.38 (m, 4H, -OC-CH_2_-), 1.84–1.70 (m, 4H, -CH_2_-); ^13^C NMR (100 MHz, CDCl_3_) *δ* 172.0, 167.8, 137.1, 129.3, 128.3, 126.3, 101.9, 98.7, 81.9, 69.9, 68.8, 63.0, 62.5, 55.4, 53.6, 33.4, 24.0 LC-MS m/z calculated for C_38_H_49_N_2_O_16_ [M + H]^+^ 789.30, found 789; HRMS (ESI+) calculated for C_38_H_48_N_2_O_16_Na [M + Na]^+^: 811.2896, found 811.2895 (Supplementary Materials)

Synthesis of compound **6.** Sebacic acid (0.0174 g, 0.086 mmol, 1 equiv.) followed by DIEA (0.065 mL, 0.38 mmol, 2 equiv.) and 2 mL of anhydrous acetonitrile were added in a 50 mL round bottom flask and the reaction mixture was allowed to stir at room temperature for 25 min. After this, compound **4** (0.0762 g, 0.19 mmol, 2.2 equiv.) was added along with 2.5 mL of anhydrous acetonitrile. The reaction mixture was left to stir for 24 h at 75 °C. Then, the rection was stopped and solvent was removed under reduced pressure and mixture was diluted using 60 mL DCM and washed with water. The combined organic layer was dried over Na_2_SO_4_ (anhydrous), filtered, and concentrated to give the crude product, which was purified using column chromatography using eluent from pure DCM to 3% MeOH/DCM to afford a white solid (65.6 mg, 90%) as the desired product. (R_f_ = 0.4 in 5% MeOH/DCM). mp 181.0–183.0 °C. ^1^H NMR (400 MHz, CDCl_3_) *δ* 7.53–7.45 (m, 4H, Ph-H), 7.42–7.31 (m, 6H, Ph-H), 6.40 (d, *J* = 8.9 Hz, 2H, NH), 5.56 (s, 2H, Ph-CH-), 4.73 (d, *J* = 3.8 Hz, 2H, H-1), 4.66–4.53 (m, 4H, -CH_2_-O), 4.34–4.20 (m, 4H, H-6a, H-2), 3.92 (t, *J* = 9.6 Hz, 2H, H-3), 3.84–3.74 (m, 4H, H-5, H-6b), 3.63–3.55 (m, 2H, H-4), 3.41 (s, 6H, OCH_3_), 2.42 (t, *J* = 7.4 Hz, 4H, -OC-CH_2_-), 1.70–1.62 (m, 4H, -CH_2_-), 1.38–1.31 (m, 8H, -CH_2_-CH_2_- ); ^13^C NMR (100 MHz, CDCl_3_) *δ* 172.2, 168.1, 137.1, 129.2, 128.3, 126.3, 102.0, 98.7, 81.9, 70.2, 68.8, 62.8, 62.5, 55.4, 53.6, 33.9, 28.8, 28.8, 24.7. LC-MS m/z calculated for C_42_H_57_N_2_O_16_ [M + H]^+^ 845.36, found 845.3; HRMS (ESI+) calculated for C_42_H_56_N_2_O_16_Na [M + Na]^+^: 867.3522, found 867.3505.

Synthesis of compound **7**. To a 50 mL round bottom flask, 4,4′-(cyclopent-1-ene-1,2-diyl)bis(5-methylthiophene-2-carboxylic acid) [18]. (50 mg, 0.14 mmol, 1.0 equiv.) was taken and dissolved in 4.0 mL of EtOH and KOH (15.3 mg, 0.27 mmol, 1.9 equiv.). The mixture was sonicated for 30 min and EtOH was removed under reduced pressure. To the flask, DMF 3.0 mL was added along with DIEA (0.07 mL, 0.43 mmol, 3.0 equiv.). The reaction mixture was stirred for 15 min and compound **4** (126.9 mg, 0.31 mmol, 2.2 equiv.) was added. The reaction was heated for 5 h at 60 °C. The reaction was stopped and DMF was dried under reduced pressure. The crude product obtained was purified further using column chromatography (DCM to 1% MeOH DCM) to obtain brown solid (113 mg, 79%) as the desired compound. R_f_: 0.4 (5% MeOH/DCM). mp 153.0–155.0 °C ^1^H NMR (400 MHz, CDCl_3_) *δ* 7.54 (s, 2H, thiophenyl-H), 7.51–7.46 (m, 4H, Ph-H), 7.38–7.33 (m, 6H, Ph-H), 6.50 (d, *J* = 8.9 Hz, 2H, NH), 5.55 (s, 2H, Ph-CH-), 4.77–4.68 (m, 6H, H-1, OCH_2_-), 4.30–4.20 (m, 4H, H-6a, H-2), 3.94 (t, *J* = 9.6 Hz, 2H, H-3), 3.84–3.72 (m, 4H, H-5, H-6b), 3.58 (t, *J* = 9.1 Hz, 2H, H-4), 3.37 (s, 6H, OCH_3_), 2.81 (t, *J* = 7.5 Hz, 4H, -CH_2_-), 2.14–2.05 (m, 2H, -CH_2_-), 2.02 (s, 6H, CH_3_); ^13^C NMR (400 MHz, CDCl_3_) *δ* 167.9, 160.4, 143.9, 137.1, 136.9, 135.6, 135.0, 129.2, 128.3, 127.7, 126.3, 101.9, 98.8, 81.8, 70.2, 68.8, 63.2, 62.5, 55.5, 53.7, 38.4, 22.9, 14.9. HRMS (ESI+) *m*/*z* calcd for C_49_H_54_N_2_O_16_S_2_Na [M + Na]^+^: 1013.2807, found 1013.2800.

Synthesis of compound **8**. To a 50 mL flame-dried flask, 4-4′-(perfluorocyclopent-1-ene-1,2-diyl)bis(5-methylthiophene-2-carboxylic acid) [42] (50 mg, 0.11 mmol, 1.0 equiv.) was dissolved in 2 mL EtOH and KOH (12.3 mg, 0.22 mmol, 2 equiv.). The mixture was sonicated for 30 min and EtOH was removed under reduced pressure. DMF 3.0 mL was added to the flask along with DIEA (0.06 mL, 0.33 mmol, 3 equiv.). The reaction mixture was stirred for 15 min and compound **4** (96.95 mg, 0.24 mmol, 2.2 equiv.) was added and left to stir for 5 h at 60 °C. Then, the reaction was stopped, and DMF was removed under reduced pressure. The reaction mixture was diluted with 5 mL ethyl acetate and washed with ice-cold water 10 mL (5 mL × 2). The organic layer was dried using anhydrous sodium sulfate and removed under reduced pressure. Column chromatography was performed using 0–3% MeOH/DCM to afford a yellowish solid (106 mg, 88.3% yield) as the desired compound. (R_f_ = 0.3 in 5% MeOH/DCM). m.p. 158.0–160.0 °C; ^1^H NMR (400 MHz, CDCl_3_) *δ* 7.79 (s, 2H, thiophenyl-H), 7.51–7.48 (m, 4H, Ph-H), 7.39–7.34 (m, 6H, Ph-H), 6.38 (d, *J* = 9.0 Hz, 2H, NH), 5.56 (s, 2H, Ph-CH-), 4.80 (d, *J* = 15.3 Hz, 2H, -CH_2_-a), 4.76 (d, *J* = 15.3 Hz, 2H, -CH_2_-b), 4.75 (d, 2H, *J* = 3.8 Hz, H-1), 4.30–4.24 (m, 4H, H-6a, H-2), 3.97–3.90 (~dt, *J* = 9.6, 3.3 Hz, 2H, H-3), 3.85–3.74 (m, 4H, H-5, H-6b), 3.58 (t, *J* = 9.1 Hz, 2H, H-4), 3.38 (s, 6H, OCH_3_), 2.81 (d, *J* = 3.3 Hz, 2H, 3-OH), 2.05 (s, 6H, CH_3_); ^13^C NMR (400 MHz, CDCl_3_) *δ* 167.3, 159.6, 149.7, 137.0, 134.0, 130. 5, 129.3, 128.3, 126.3, 125.7, 102.0, 98.7, 81.8, 70.4, 68.5, 63.5, 62.5, 55.4, 53.6, 15.0. HRMS (ESI+) *m*/*z* calcd for C_49_H_48_N_2_F_6_O_16_S_2_Na [M + Na]^+^: 1121.2242, found 1121.2233.

## Figures and Tables

**Table 1:** Gelation properties of the dimeric glycolipids.

Cpd. #	Hex	Tol	n-BuOH	i-PrOH	EtOH	EG	EtOH :H_2_O (1:1)	EtOH :H_2_O (1:2)	DMSO: H_2_O (1:1)	DMSO: H_2_O (1:2)	H_2_O
**5**	I	I	G5.0_T_	PG	PG	G20.0_C_	G10.0_T_	PG	G6.7_T_	G5.0_T_	P
**6**	P	I	G2.8_T_	G5.0_T_	G10.0_T_	G6.7_C_	G4.0_T_	G4.0_T_	G5.0_O_	G4.0_T_	I
**7**	I	G10_T_	G6.6_O_	G6.6_O_	G4.0_T_	G6.6_T_	G10_O_	G20_O_	G1.6_O_	G1.6_O_	I
**8**	I	P	G20_O_	P	G10_T_	G10_T_	P	P	P	P	I

All compounds were tested starting from 20 mg/mL. The numbers are minimum gelation concentrations (MGCs) in mg/mL; G, stable gel at room temperature; P, precipitation; S, soluble; I, insoluble; PG, partial gel at 20 mg/mL; T, translucent; C, clear; O, opaque. Hex—hexane, EG—ethylene glycol, Tol—toluene. All compounds were insoluble in hexane, water at 20 mg/mL.

## Data Availability

Not applicable.

## Supplementary Materials

The following are available online at https://www.mdpi.com/article/10.3390/molecules28176228/s1, ESI includes (1) ^1^H and ^13^C NMR spectra of compounds **5**–**8** and 2D NMR spectra of compounds **7**–**8**; (2) dye absorption studies and additional gel column photos.
