# Supplementary material for: Synthesis and Self-Assembling Properties of Carbohydrate- and Diarylethene-Based Photoswitchable Molecular Gelators
Source: Molecules. 2023 Aug 24;28(17):6228. doi: 10.3390/molecules28176228 (PMC10488493; doi:10.3390/molecules28176228)
Supplement: Supplementary file 1 [file molecules-28-06228-s001.zip › molecules-2543999-supplementary.pdf]

Supporting Information

Synthesis and Self-Assembling Properties of Carbohydrate and Diarylethene Based Photoswitchable Molecular Gelators

Pramod Aryal, Joedian Morris, Surya Adhikari, Jonathan Bietsch, Guijun Wang* Department of Chemistry and Biochemistry, Old Dominion University, Norfolk, VA 23529, USA, g1wang@odue.edu

Table of contents……………………….…………………......………………………….S1

^{1}H, ^{13}C, and 2D NMR spectra for compounds **5-8**.….…………..……………………..…...S2

Gel stablity under acidic and basic conditions……………………………………………S10

Dye removal experiments…………….……………………………………………………….S17

FTIR spectra for compounds **5-8**…..…….……………………………………..……...…..S25

I. ^{1}H, ^{13}C NMR and 2D NMR spectra

(Bruker 400 MHz NMR instrument for all).

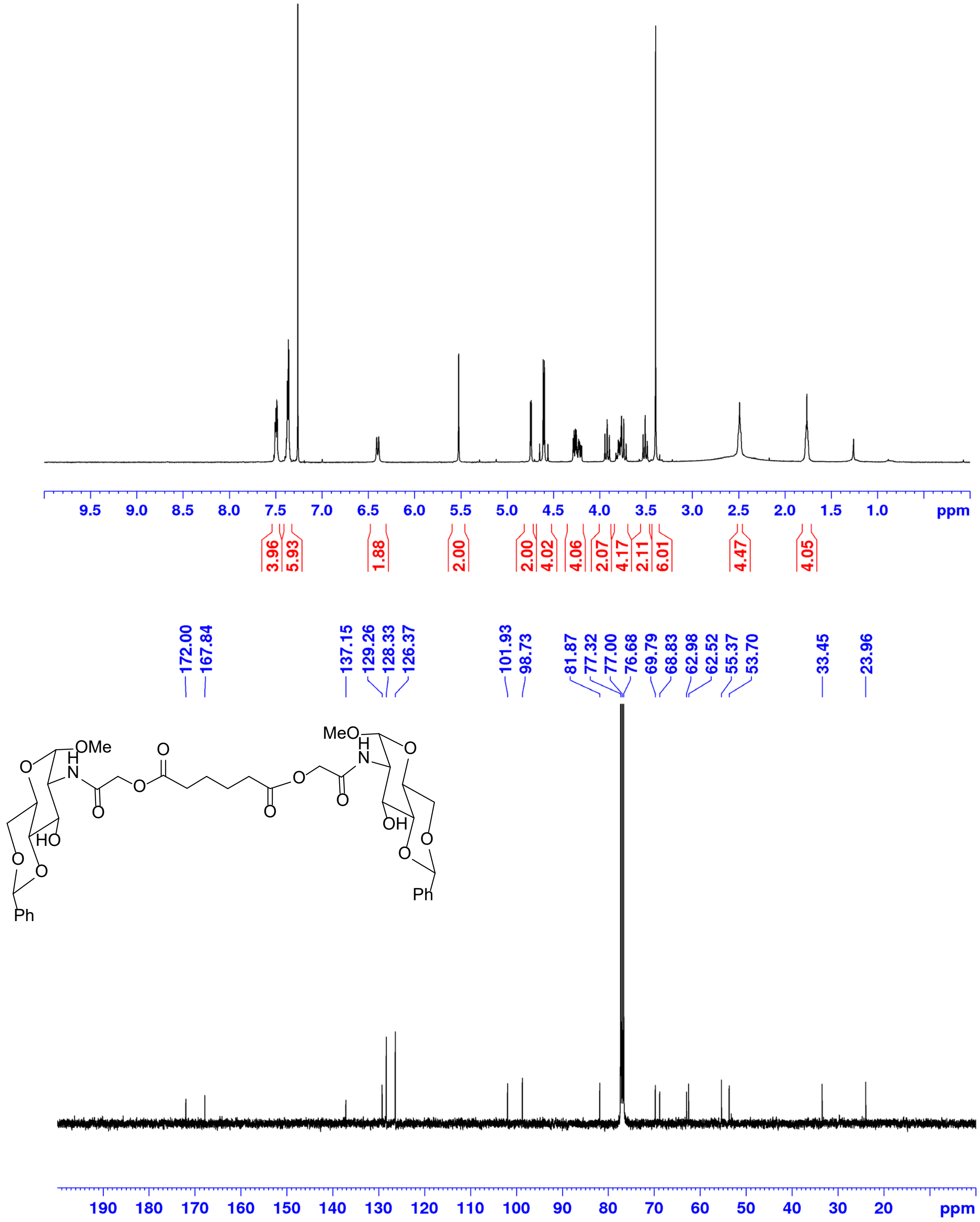

Figure S1. ^{1}H and ^{13}C NMR spectra of compound **5** in $CDCl_3$

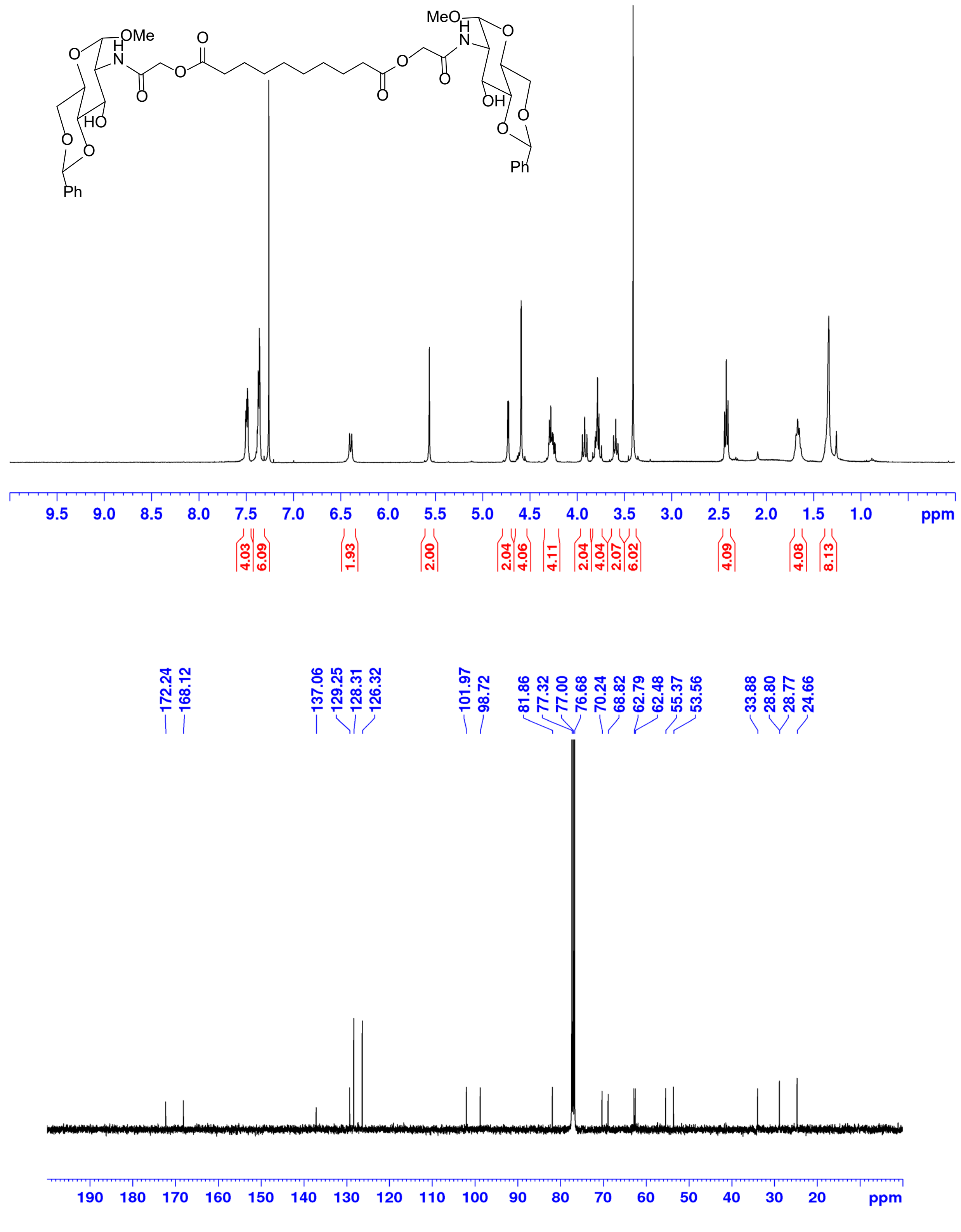

Figure S2. ^{1}H and ^{13}C NMR spectra of compound **6** in $CDCl_3$.

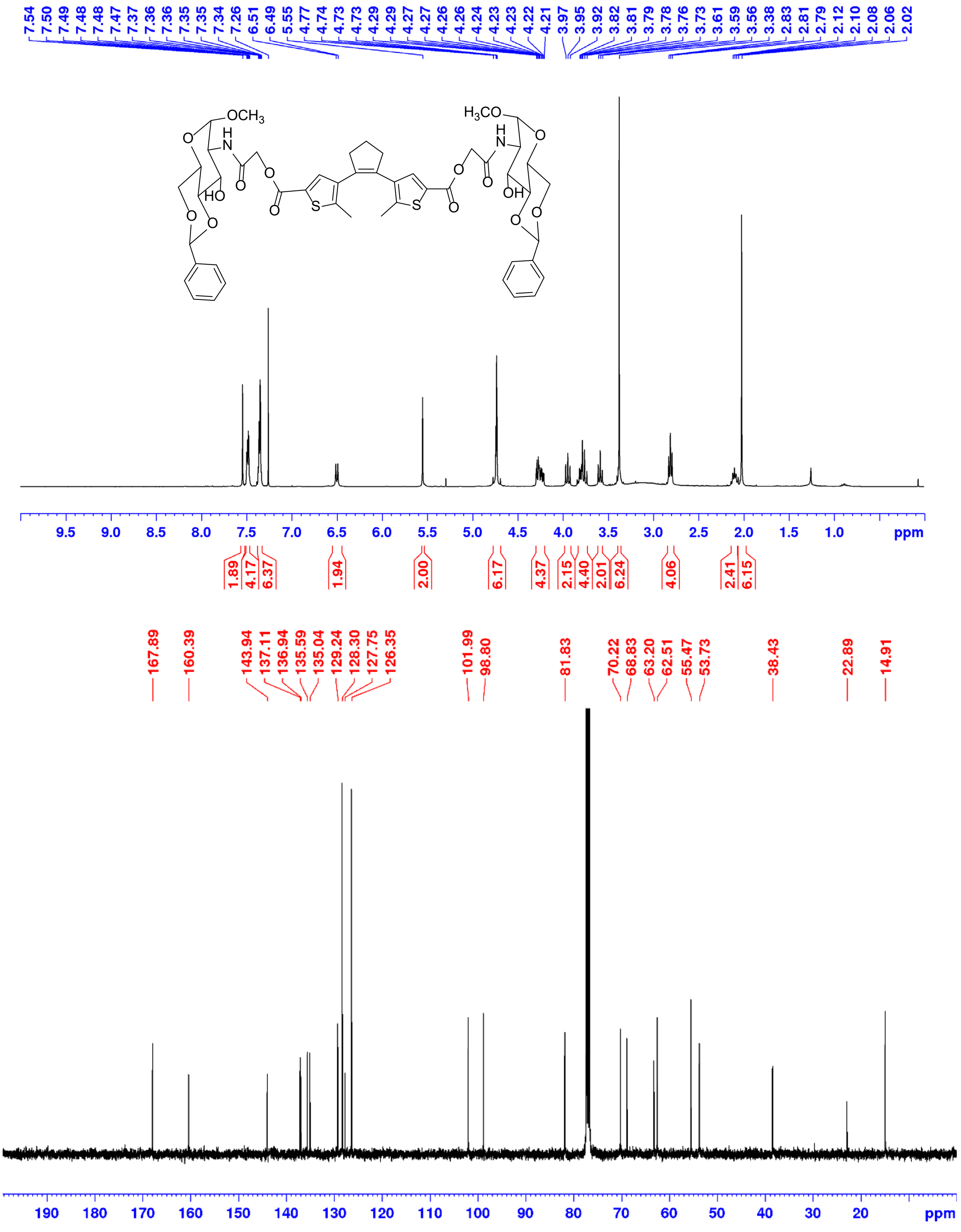

Figure S3. ^{1}H and ^{13}C NMR spectra of compound **7** in $CDCl_3$.

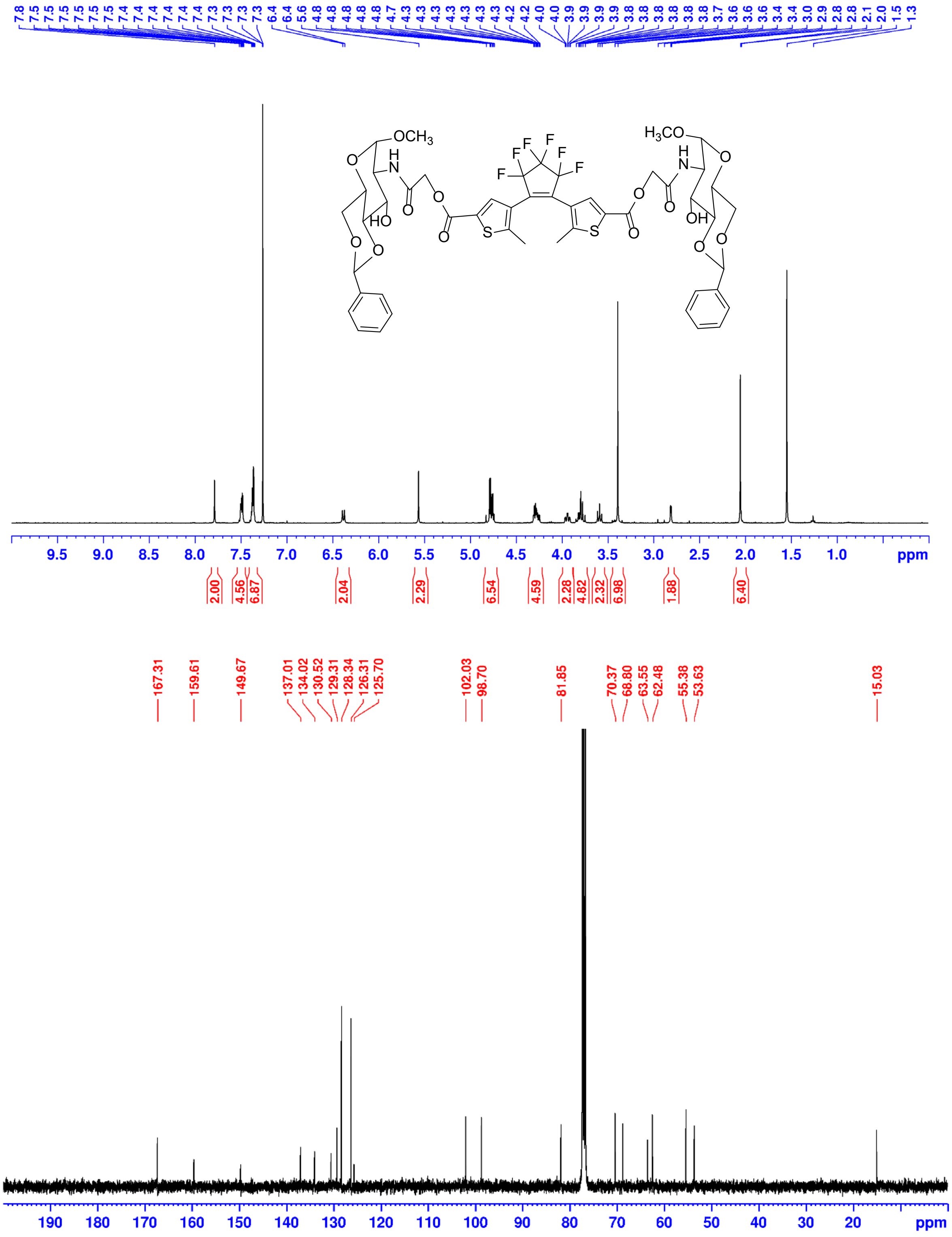

Figure S4. ^{1}H and ^{13}C NMR spectra of compound **8** in $CDCl_3$.

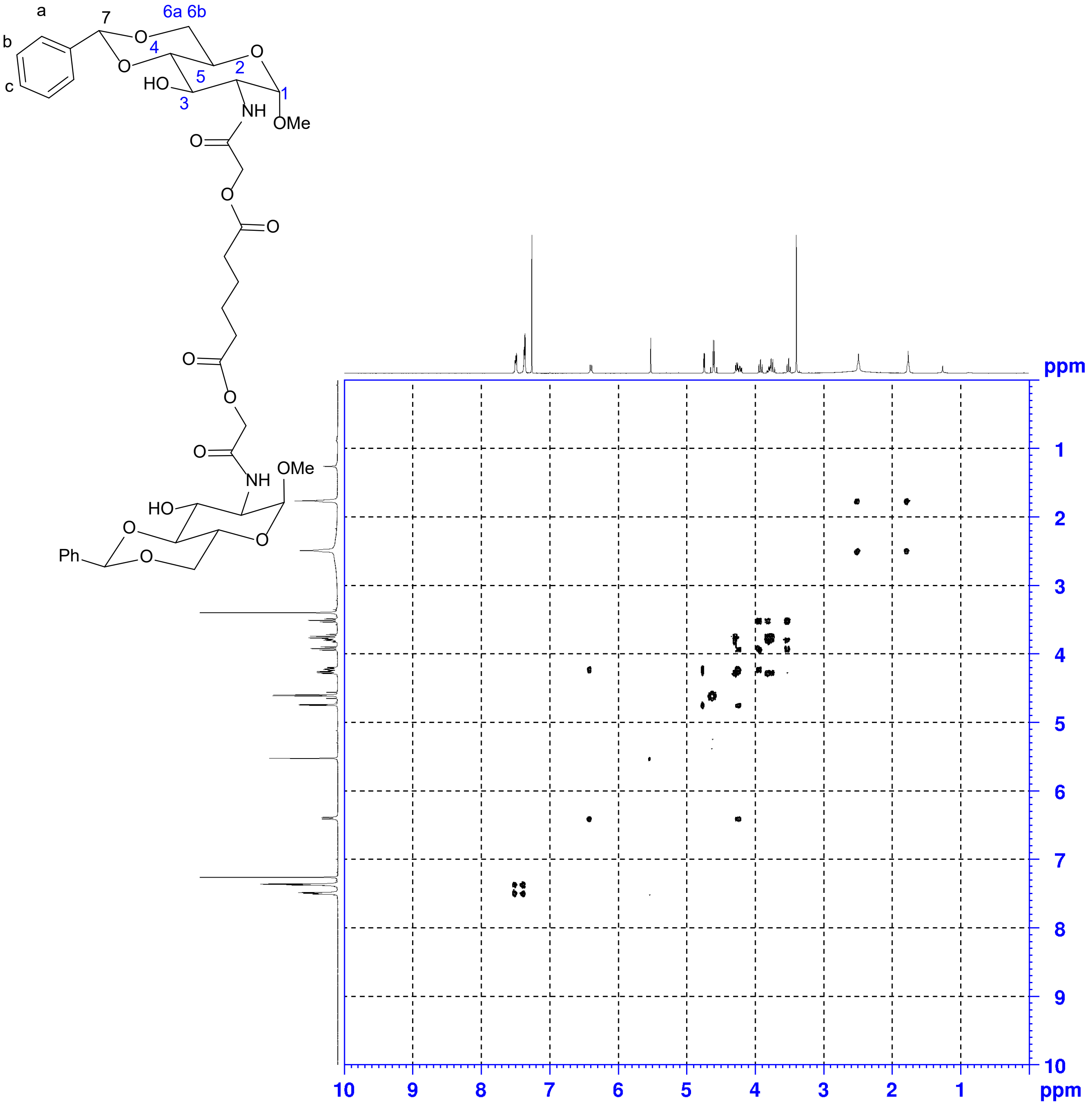

Figure S5a. The structure of compound **5** with hydrogen positions labelled and its COSY NMR spectra.

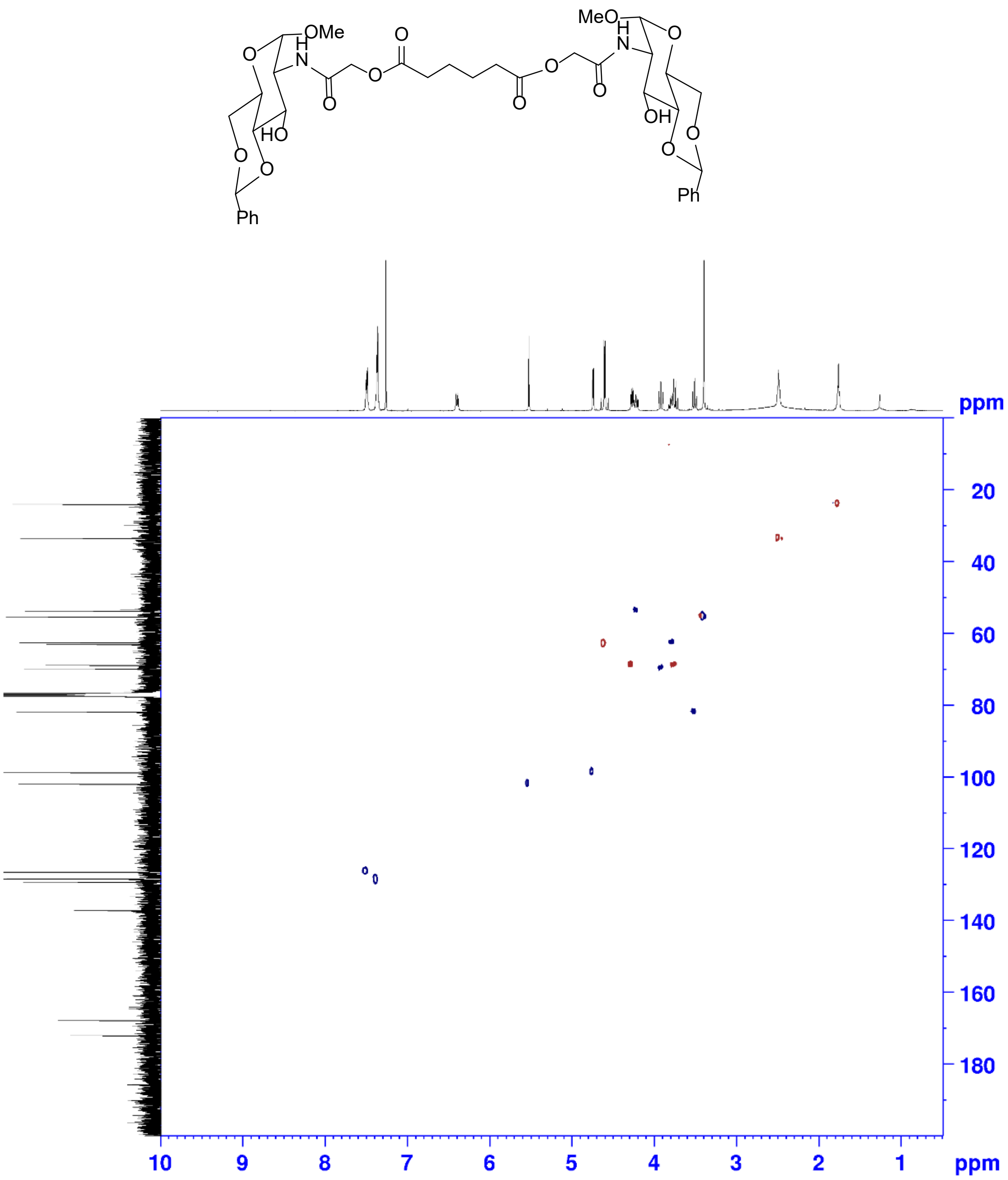

Figure S5b. HSQC NMR spectra of compound **5** in $CDCl_3$.

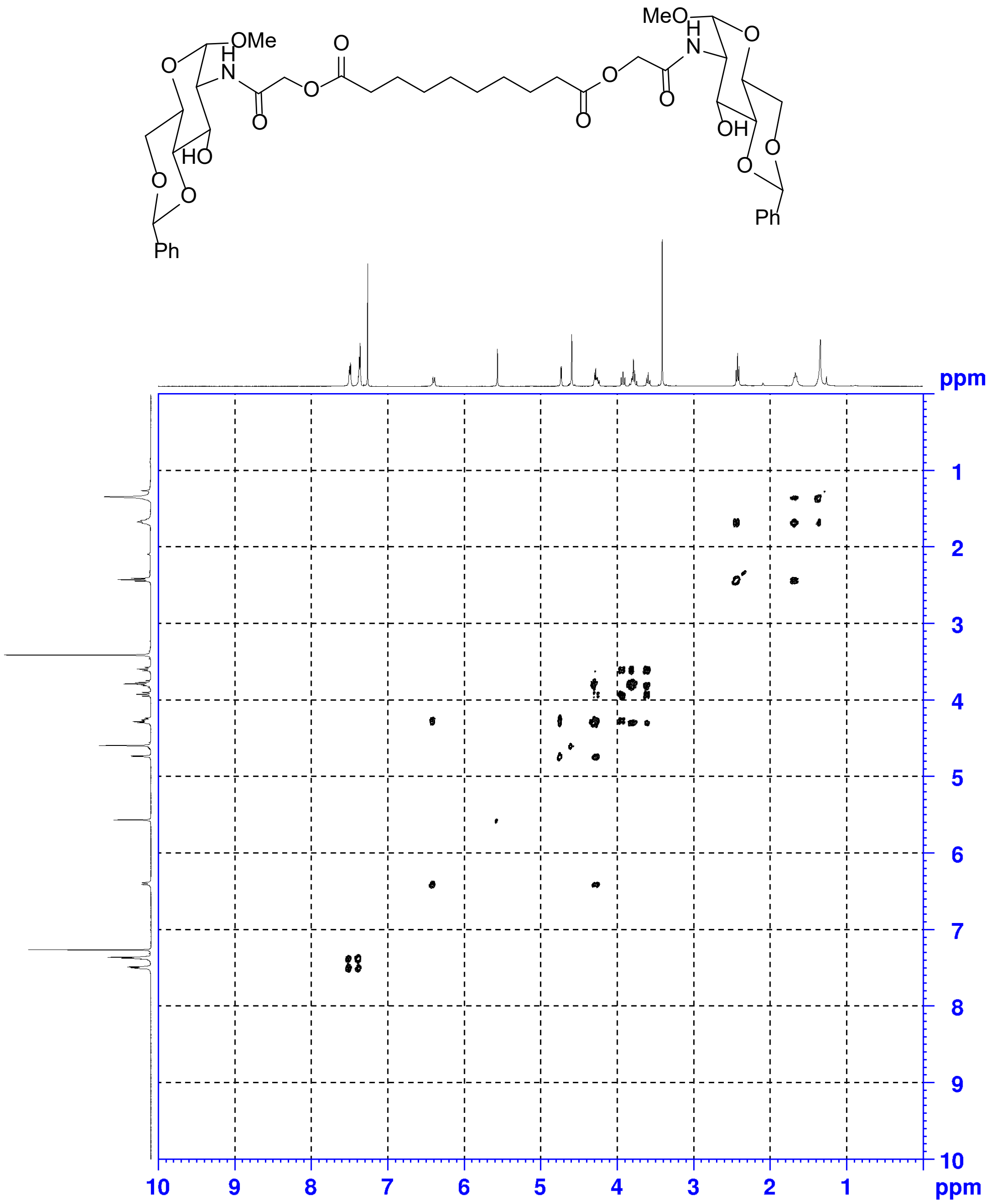

Figure S6a. COSY NMR spectra of compound **6** in $CDCl_3$.

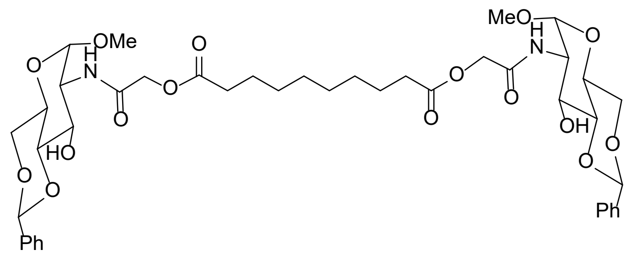

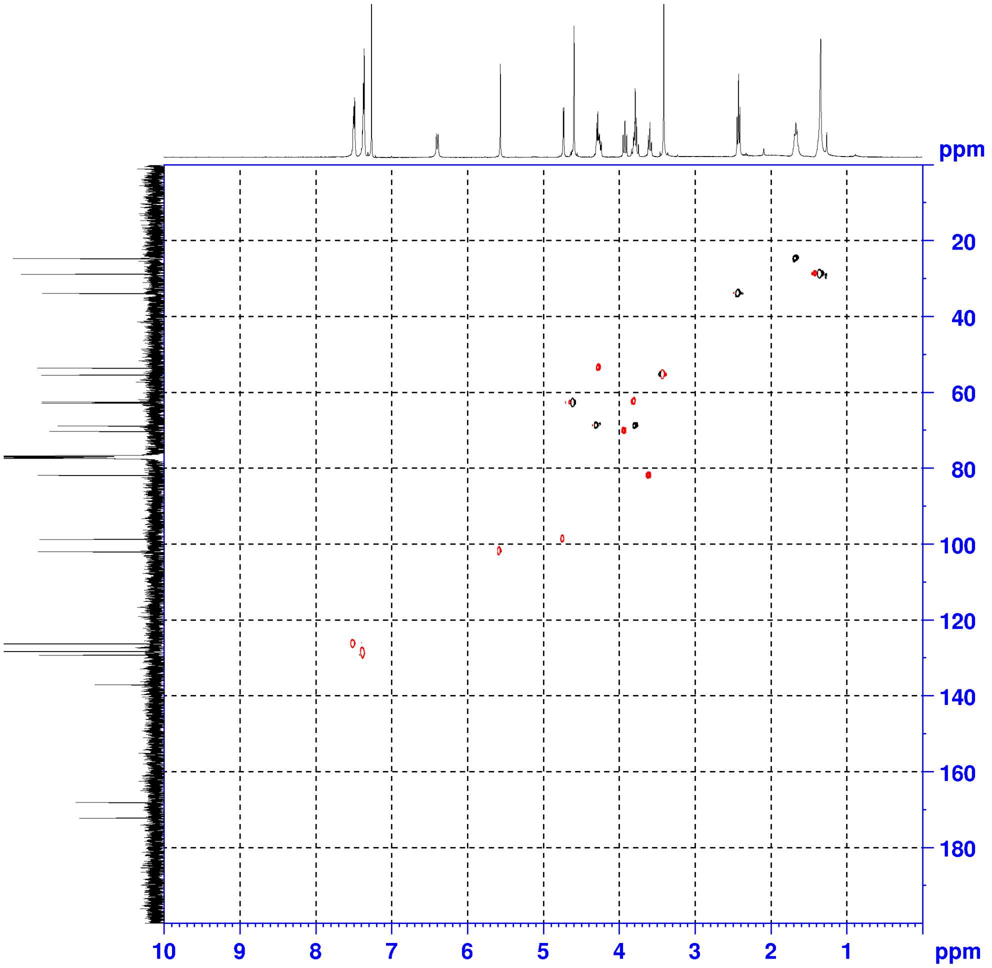

Figure S6b. HSQC NMR spectra of compound **6** in $CDCl_3$.

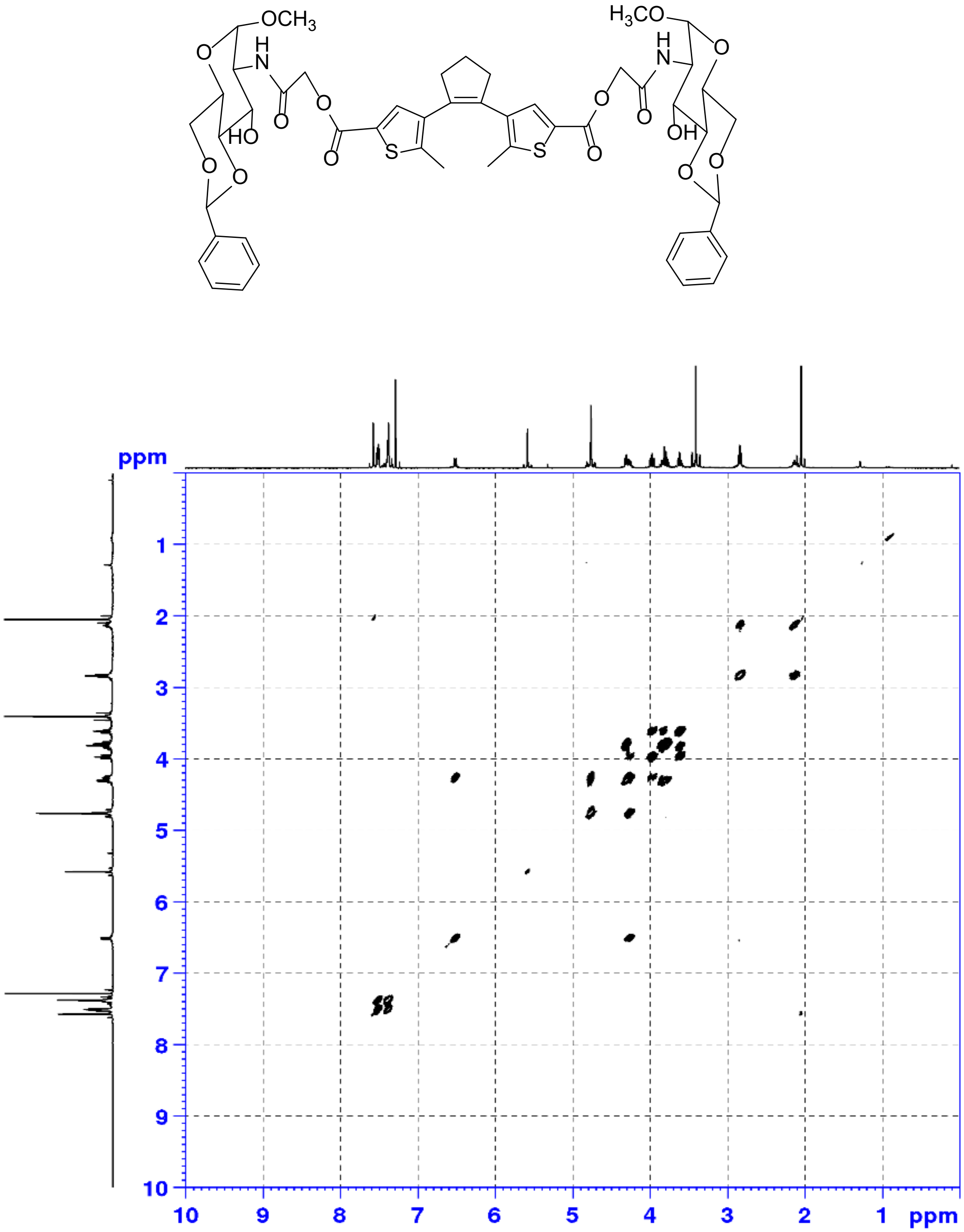

Figure S7a. 2D COSY NMR spectrum for compound **7** in $CDCl_3$.

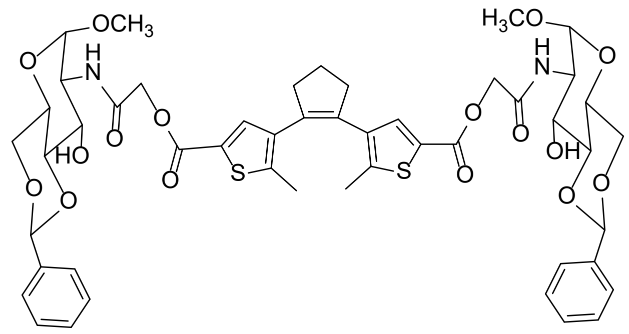

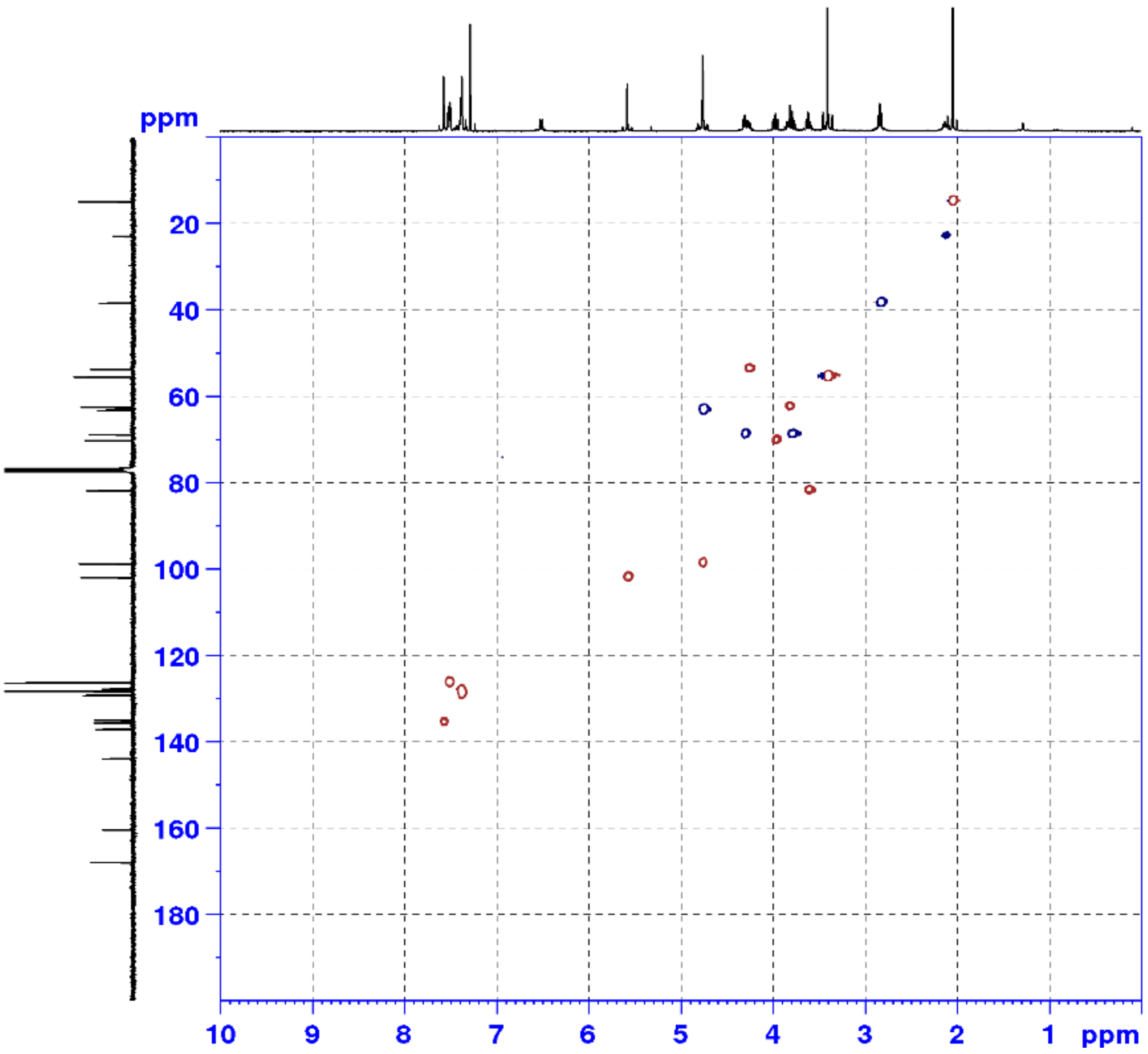

Figure S7b. 2D HSQC NMR spectra of compound **7** in $CDCl_3$.

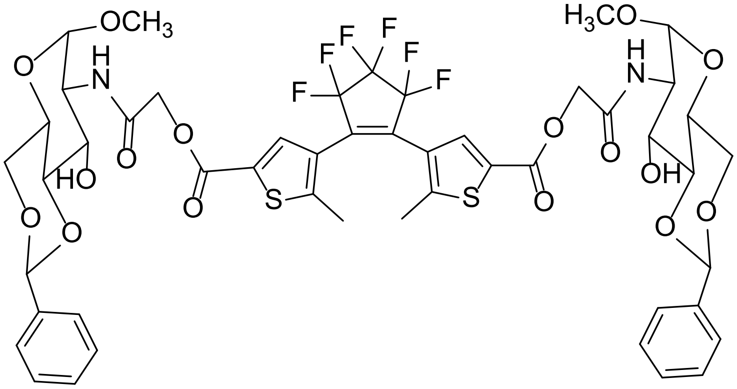

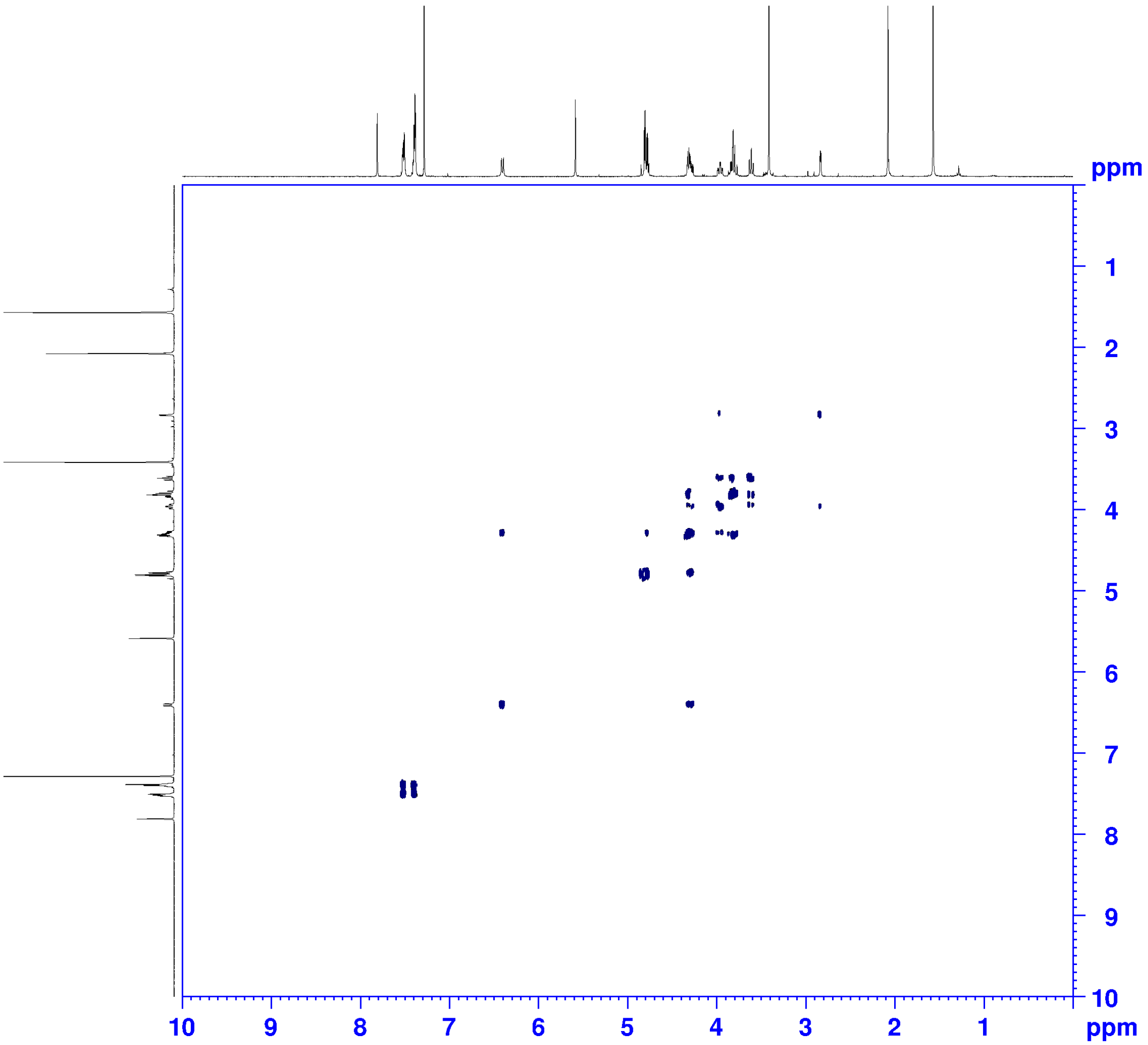

Figure S8a. 2D COSY NMR spectrum for compound **8** in $CDCl_3$.

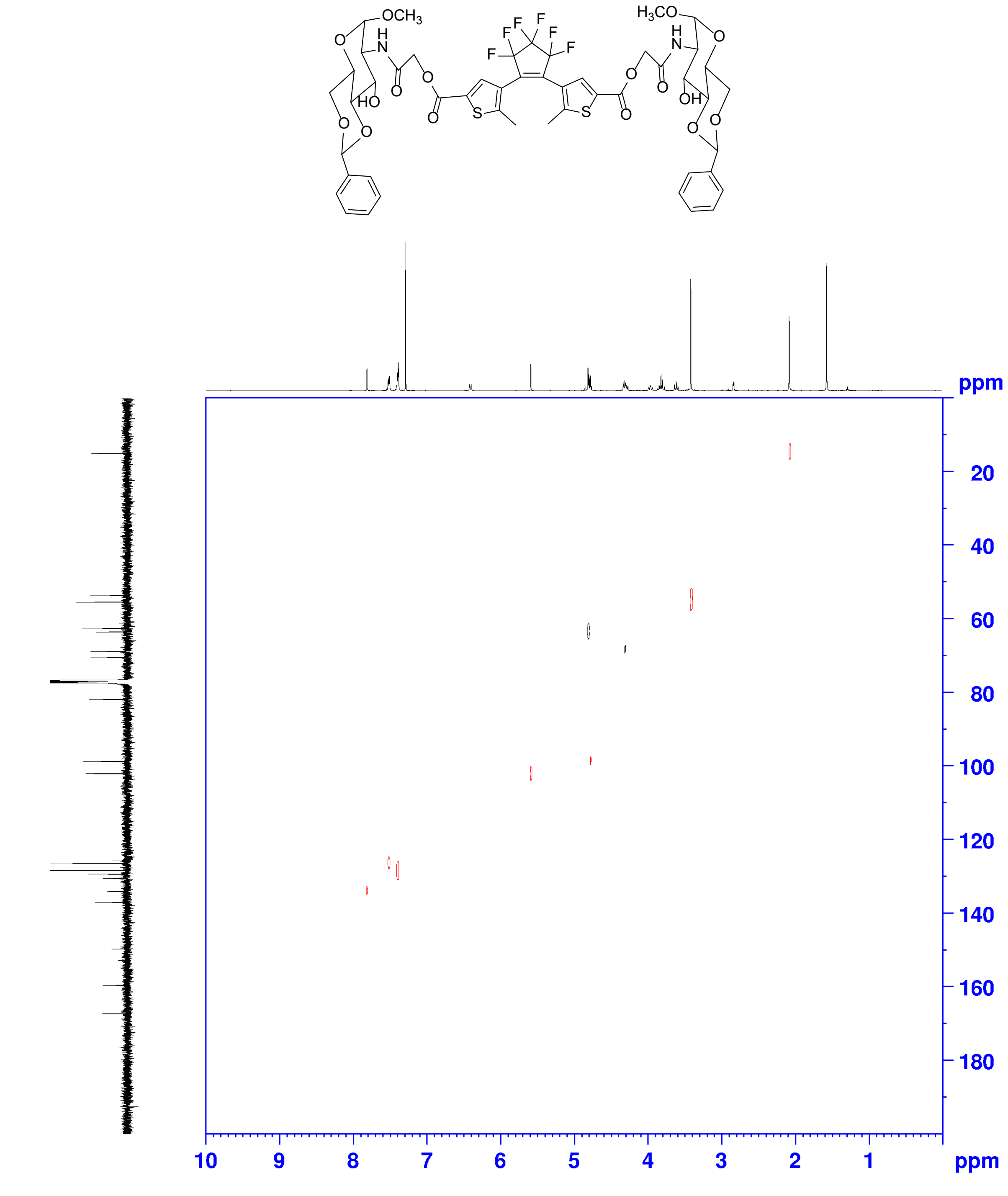

Figure S8b. 2D HSQC NMR spectra of compound **8** in $CDCl_3$.

II. Stabiliy of gels under basic and acidic conditions

1. Gel stablility under basic conditions

In a one dram vial, 4.0 mg of compound **5** was mixed with 0.5 mL of DMSO:H_2O (v/v 1:1) to make a gel (two gels were made). After 2 hours, 0.5 mL of pH 12 solution was added to the vials and the gels were observed every hour to record any visible decomposition that occurred, the gels appeared stable at 5 hours and started to become unstable as time goes on. The gel vial images are shown in Figures S9. After the gels fully disintegrated, the resulting compound was extracted with 2 mL of DCM and a proton NMR spectrum was obtained and included in Figures S11 and S12.

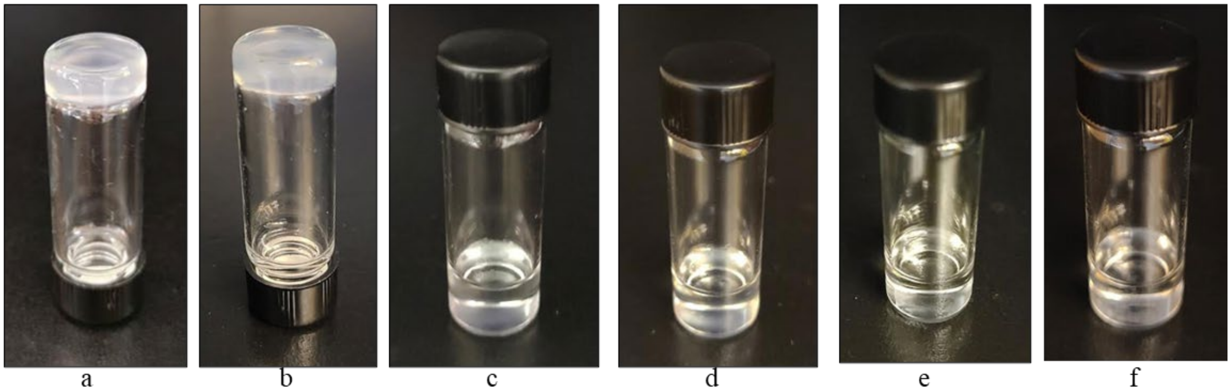

a b c d e f

Figure S9. Pictures of gels of compound **5** under basic conditions at pH 12. a) Inverted gel vial at pH 12 at 0 hour; (b) Inverted gel vial at pH 12 at 5 hours; (c) The vial at pH 12 at 22 hours; (d) The vial at pH 12 at 26 hours; (e) The vial at pH 12 at 46 hours; (f) The vial at pH 12 at 120 hours.

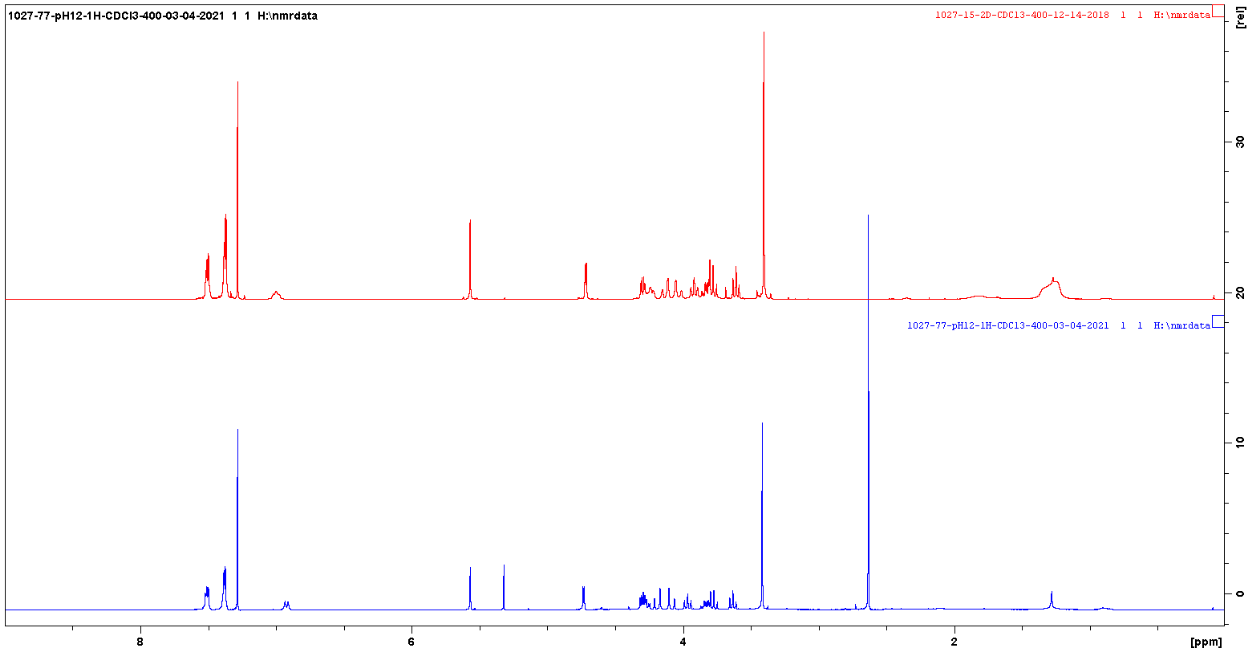

Figure S10. Overlay of ^{1}H NMR spectrum before (top) and after (bottom) base treatment of compound **5** at pH 12. The compound seems to remain the same at the end.

2. Gel stability under acid conditions

4 mg of compound **5** was added to a 1-dram vial and 0.5 mL of DMSO: H_2O (1:1) was added to make the gel (two gels were made). After 2 hours, 0.5 mL of pH 2.15 or 4.01 solution was added to the vials and the gels were observed every hour to record any visible decomposition that occurred. The observations are recorded in Table S2, the gel photos at different time points are included in Figures S11 and S12.

Table S1. Observation of pH dependent gel stability study at different time.

**Time**	**Observations pH 2.15**	**Observations pH 4.01**
0 h	Gel slightly disturbed upon the addition of the pH solution.	Gel slightly disturbed upon the addition of the pH solution but stable.
1h	Stable gel	Stable gel
2 h	Stable gel	Stable gel
4 h	Stable gel	Stable gel
5 h	Stable gel	Stable gel
23 h	Stable gel	Stable gel
47 h	Stable gel	Stable gel, top of gel a bit disturbed.
71h	Stable gel	Stable gel
94 h	Stable gel	Stable gel
144 h	Stable gel	Stable gel
7 days	Stable gel	Stable gel
10 days	Stable gel	Stable gel
18 days 1h	Relatively Stable	Relatively Stable

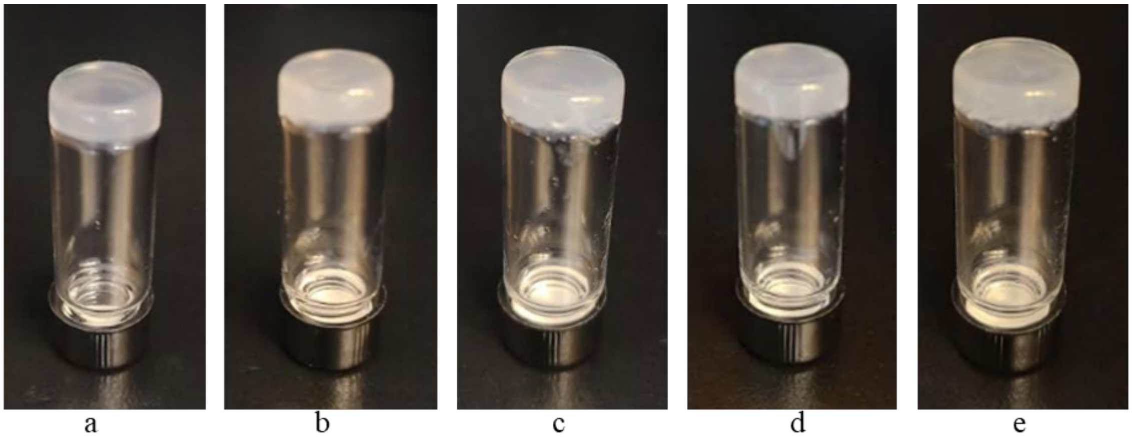

Figure S11. Pictures of gels of compound **5** under acidic conditions. a) Inverted gel vial at pH 2.15 at 0 hour; (b) Inverted gel vial at pH 2.15 at 2 hours; (c) Inverted gel vial at pH 2.15 at 5 hour; (d) Inverted gel vial at pH 2.15 at 23 hour; (e) Inverted gel vial at pH 2.15 at 47 hour.

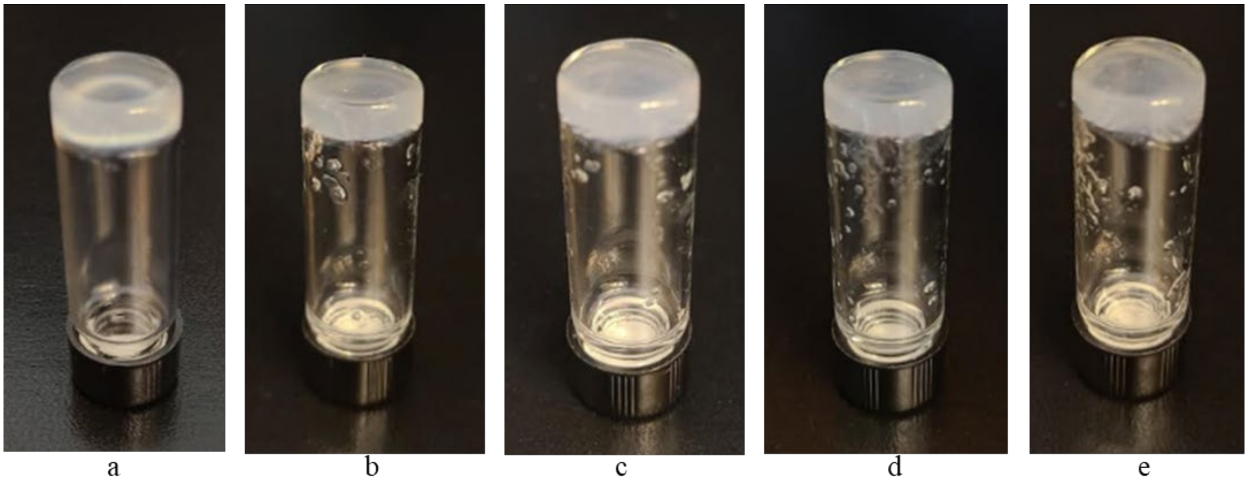

Figure S12. Pictures of gels of compound **5** under acidic conditions. (a) Inverted gel vial at pH 4.01 at 0 hour; (b) Inverted gel vial at pH 4.01 at 2 hours; (c) Inverted gel vial at pH 4.01 at 5 hour; (d) Inverted gel vial at pH 4.01 at 23 hour; (e) Inverted gel vial at pH 4.01 at 47 hour.

III. Dye removal experiments

1. Rhodamine B dye absorption study using compound **6**

A gel was prepared by dissolving 10 mg of compound **6** in 2.0 mL of DMSO: H_2O (1:2). The gel was allowed to sit for 1 h before starting the experiment. Then 2.0 mL of a 0.0082 mM solution of the rhodamine B base dye was added on top of the gel carefully. Time dependent UV-Vis spectra of the rhodamine B base solution above the gel are shown in Figure 7. The images of the gel photos at different time points are shown in Figure S15. The rhodamine B base standard in Figure 7 relates to the initially added 2.0 mL of a 0.0082 mM solution of the rhodamine B base dye which gives the maximum absorption. As shown in Figure S13, the gel was able to slowly absorb the dye over time. After approximately 282 h, maximum absorption of the dye was achieved by the gel. The absorbance profile of rhodamine B base by the gel formed by compound **6** for the peak at 554 nm is shown in Figure S14. The ratios shown are calculated by using 100%−(the absorbance of the

aqueous phase/maximum absorbance). The absorbance profile of the rhodamine B base that was left in the aqueous solution on top of the gel of compound **6** for the peak at 554 nm is included in Figures S15. The ratios are calculated using the absorbance of the aqueous phase/absorbance of the standard.

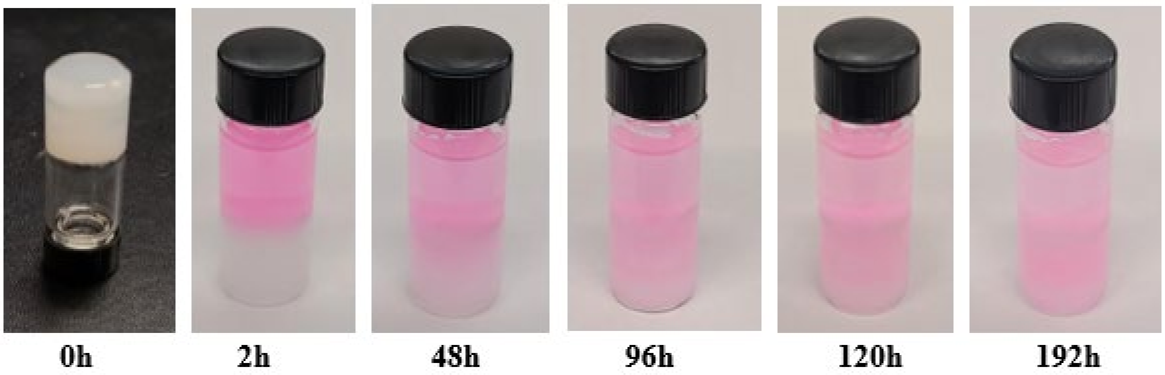

Figure S13. Gel photos at different time points. At 0 h the gel vial was inverted. After adding rhodamine B dye solution at different times are included.

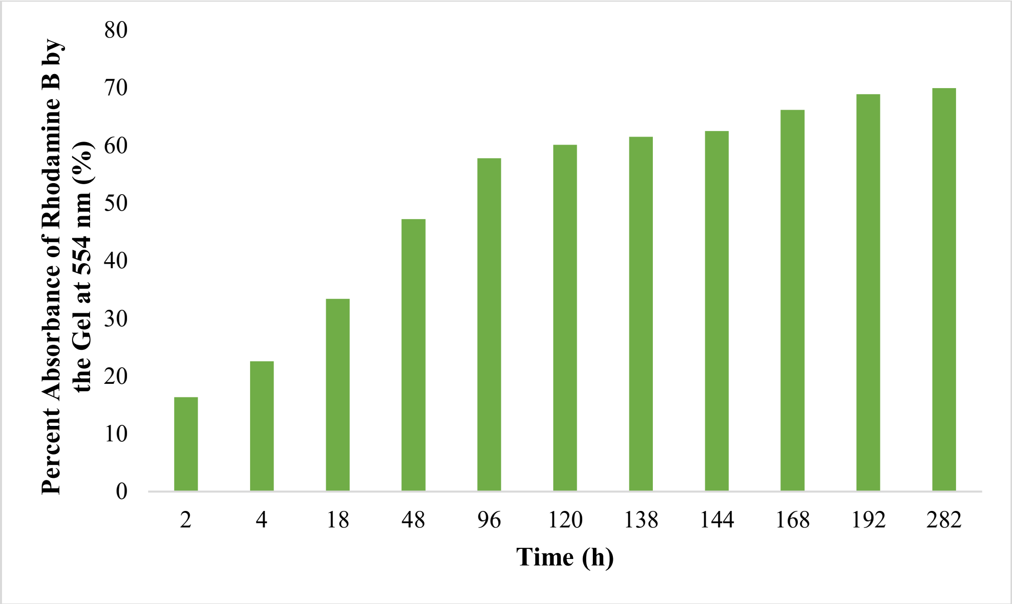

Figure 14. The absorbance profile of rhodamine B base by the gel formed by compound **6** for the peak at 554 nm in Figure 7. The ratios shown are calculated by using 100% - (the absorbance of the aqueous phase/maximum absorbance).

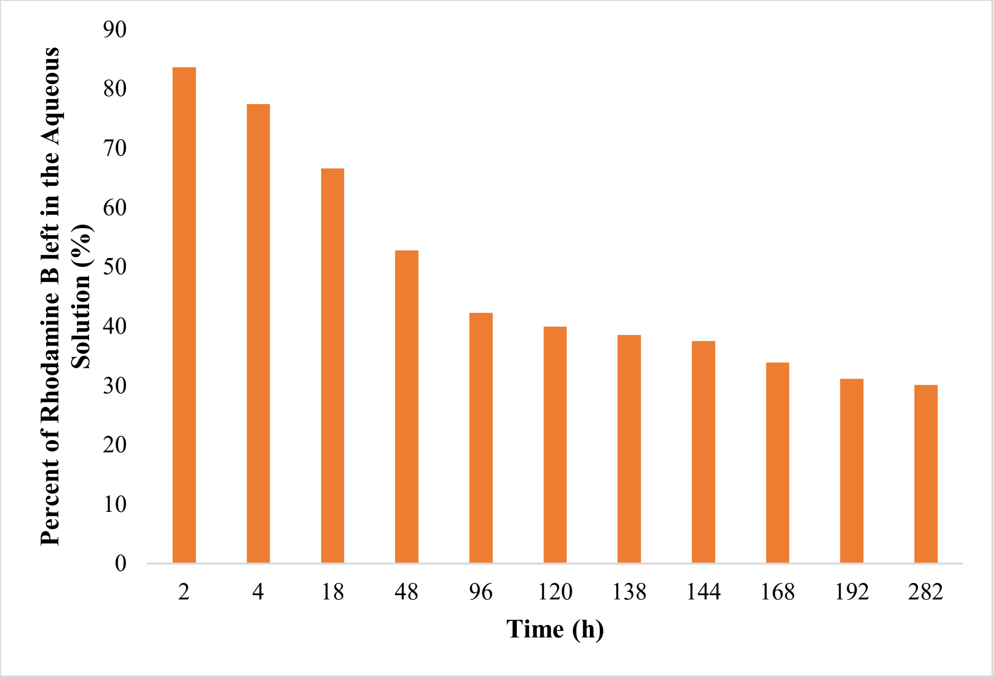

Figure 15. The absorbance the rhodamine B base that was left in the aqueous solution on top of the gel. The ratios are the absorbance of the aqueous phase/absorbance of the standard.

2. Dye removal studies using gel column filtration

2.1 Calibration curve for dyes using UV-Vis spectrometry

Rhodamine B Base (2 mg, 0.00452 mM) was dissolved in deionized water to prepare a 2.0 mg/L solution, serial dilution was performed to prepare the other solutions: 1 mg/L, 0.5 mg/L, and 0.25 mg/L solutions. UV-Vis absorption was taken for all the samples to make standard calibration curve and the absorption at 554 nm (λmax) was used to calculate the calibration curve.

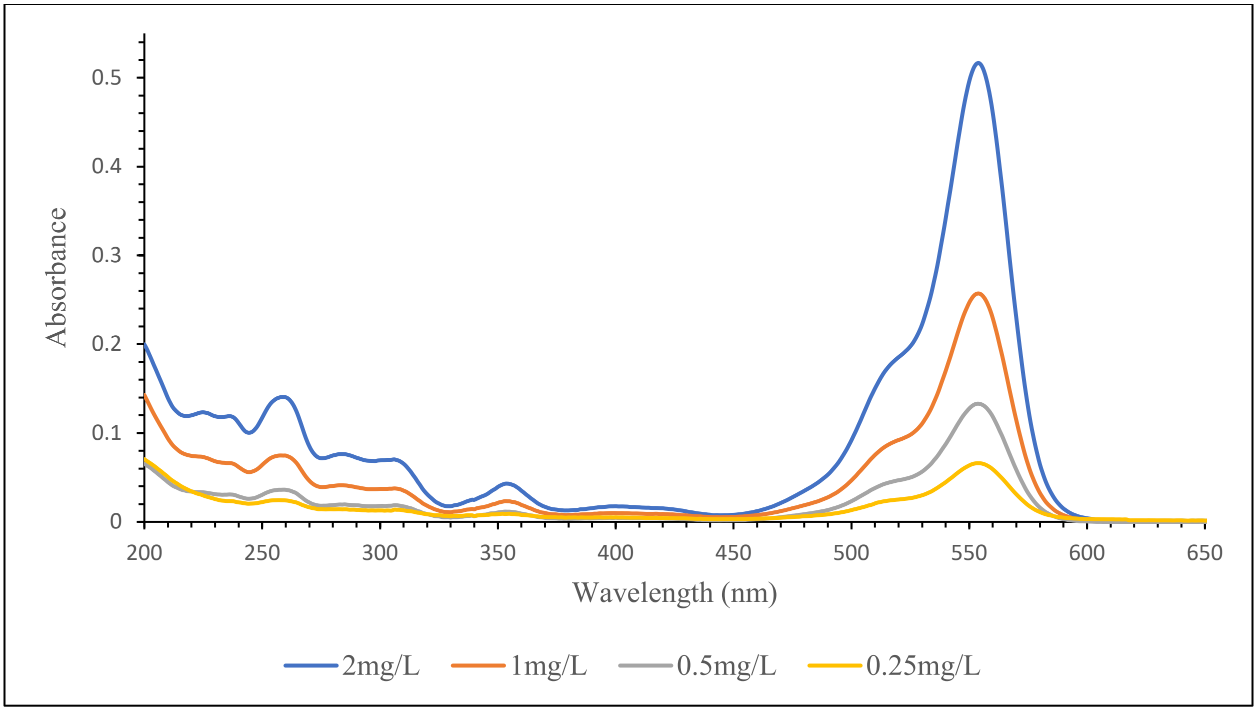

Figure S16. UV-Vis spectra obtained from a serial dilution of Rhodamine B Base.

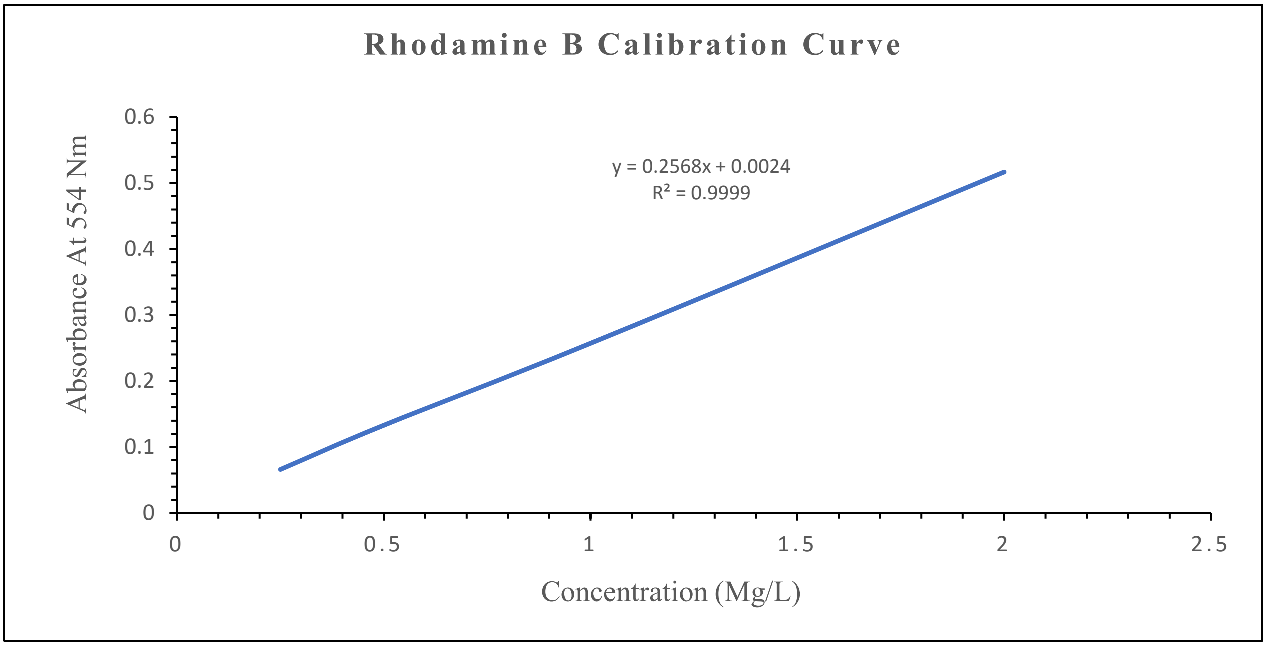

Figure S17. Calibration curve obtained from absorbance reading at 554 nm (λmax).

A dilution study was performed using toluidine blue to obtain the standard curve of the dye. An aqueous stock solution was prepared by dissolving toluidine blue (10.0 mg) in DI water in a 1 L volumetric flask to yield a concentration of 32.7 μM. This was then serial diluted by transferring 5 mL of the solution to a 10 mL volumetric flask using a volumetric pipette, then diluting it with DI water to obtain solutions with concentrations of 16.35 μM, 8.175 μM, 4.0875 μM, 0.327 μM, 0.0327 μM and 0.000 μM.

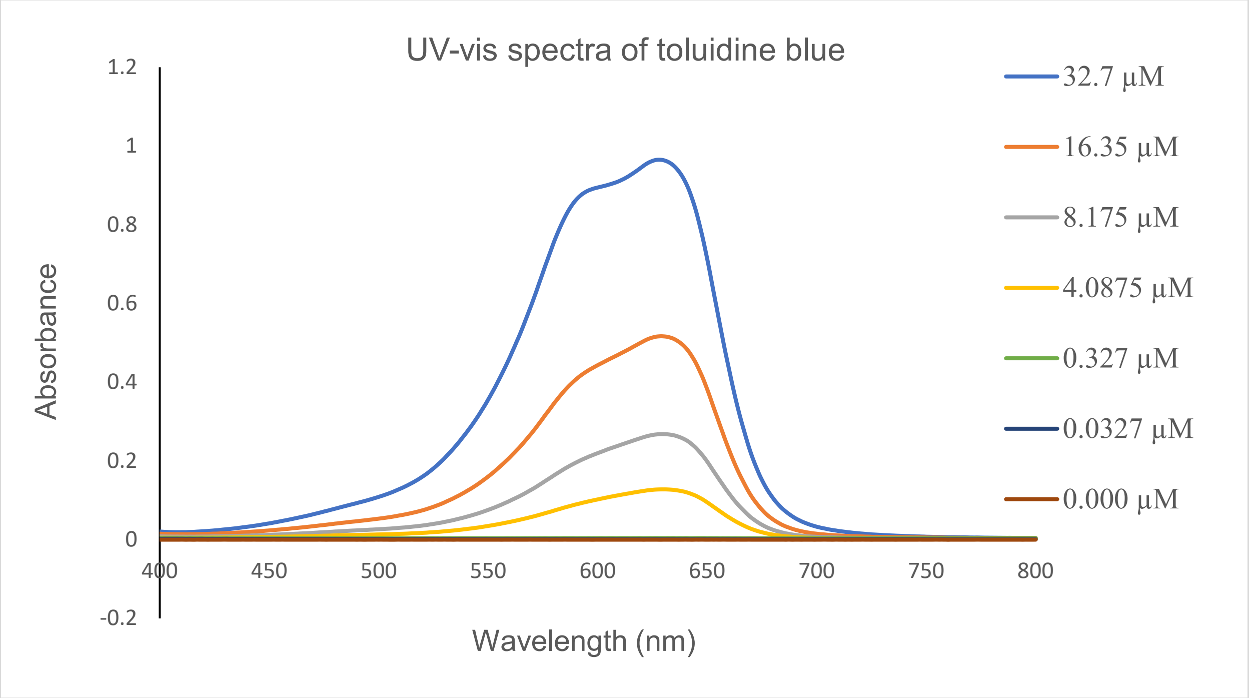

Figure S18. Various UV-Vis spectra obtained from a serial dilution of toluidine blue.

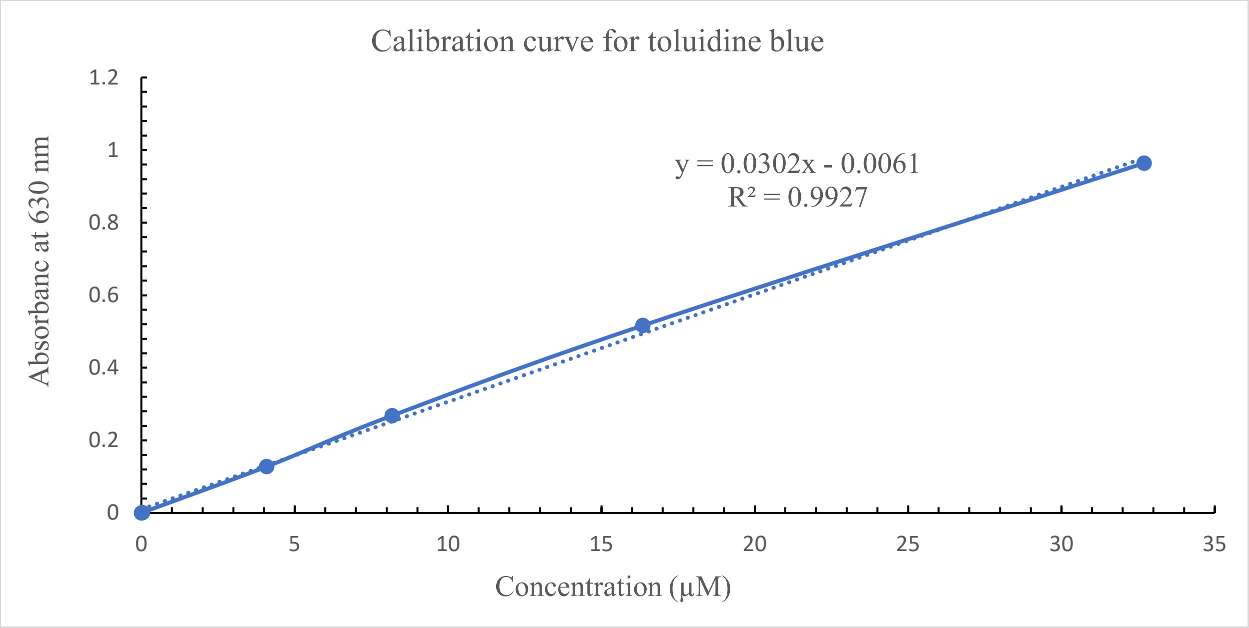

Figure S19. Calibration curve obtained from absorbance reading at 630 nm (λmax).

2.2. Toluidine blue dye absorption study using compound **7**

2.2.1. Using the gels formed by compound **7**Open

A gel (1.0 mL) formed by compound **7** in 4 mg/mL DMSO:H_2O (v/v 1:2) was evaluated for the absorption of toluidine blue dye (TBO). A solution was preparing by heating the compound **7** in the solvent, the solution was then transferred into a gel column with a sealed plug and the solution was settled in the gel column for 14 hours, a stable gel formed inside the syringe column as shown in Figure 9a in the main text. 1.0 mL DI water was added on top of the gel column and the plug was opened to elute from the gel column to displace DMSO from the gel. The column was resealed with the plug, then 0.1 mL (50 mg/L) TBO was added to the gel column and left settling for 30 min, then the dye was eluted from the gel column, after this, another 1.0 mL DI water was added to the gel column, the total eluted liquid was 1.2 mL. This was diluted to 2.2 mL and the UV-Vis

spectra were observed (Figure 8). The gel was stable enough to be inverted at the end of the experiment (Figure 9g). Absorbance at λ_{max} (630 nm) of toluidine blue was measured using a calibration curve, about 95% of the dye from the initial solution.

UV absorption λ_{max} (630 nm) = 0.004167

From calibration curve: $y = 0.0302x - 0.0061$

$$0.004167 = 0.0302X - 0.0061 \rightarrow x = \frac{0.004167 + 0.0061}{0.0302} = 0.33996\ \mu M$$

Amount of TB after column: 0.33996 μM × (1 L/1000) × 305.82 × (1 L/1000 mL) × 2.2 mL =0.0002287 mg

Original amount of TB added to column: 50.0 mg/L × (1 L/1000 mL) × 0.1 mL= 0.005 mg

Amount of TB removed from the solution: 0.00477 mg, 95%

2.2.2. Toluidine blue dye absorption study using the gels of **7**Close.

The gels formed by compound **7** cyclized form was also evaluated for the TBO dye removal. Compound **7** (4.0 mg) was mixed with 1.0 mL DMSO:H_2O (v/v 1:2) by sonication in a 1-dram vial, the cloudy solution was irradiated with a 6W UV lamp at 302 nm for 30 min. The solution turned purple, this was then heated to form a homogenous solution, and transferred to a syringe column with a plug. The syringe column was settled for 14 hours in a dark room and a stable gel formed as shown in Figure S23a. The experiment was performed in dark room and photograph was taken using with low light photography. The gel formed by **7**Close (Figure S23a) was eluted with1.0 mL DI water to flush DMSO from the gel column. Then, toluidine blue dye solution 0.1 mL (50 mg/L) was added to the gel column and allowed to settle for 30 min, after that the plug was removed and the dye solution was passing through the column. Then 1.0 mL water was added on top of the gel to elute again, a total 1.2 of liquid was collected. This liquid was diluted to 2.2

mL in order to take the UV-Vis spectrum. The gel was stable enough to be inverted at the end of the experiment as shown in Figure S23g.

The estimated amount of dye removed (absorbed by the gel column) is calculated using the calibration curve and approximately 96% of the dye was removed from the initial solution.

UV absorption λ_{max} (630 nm) = 0.003067

From calibration curve: $y = 0.0302x - 0.0061$

$$0.003067 = 0.0302X - 0.0061 \rightarrow x = \frac{0.003067 + 0.0061}{0.0302} = 0.0302\ \mu M$$

Amount of TB after column: 0.0302 μM × (1 L/1000) × 305.82 × (1 L/1000 mL) × 2.2 mL =0.000204 mg

Original amount of TB added to column: 50.0 mg/L × (1 L/1000 mL) × 0.1 mL= 0.005 mg

Amount of TB removed from the solution: 0.00479 mg, 96%

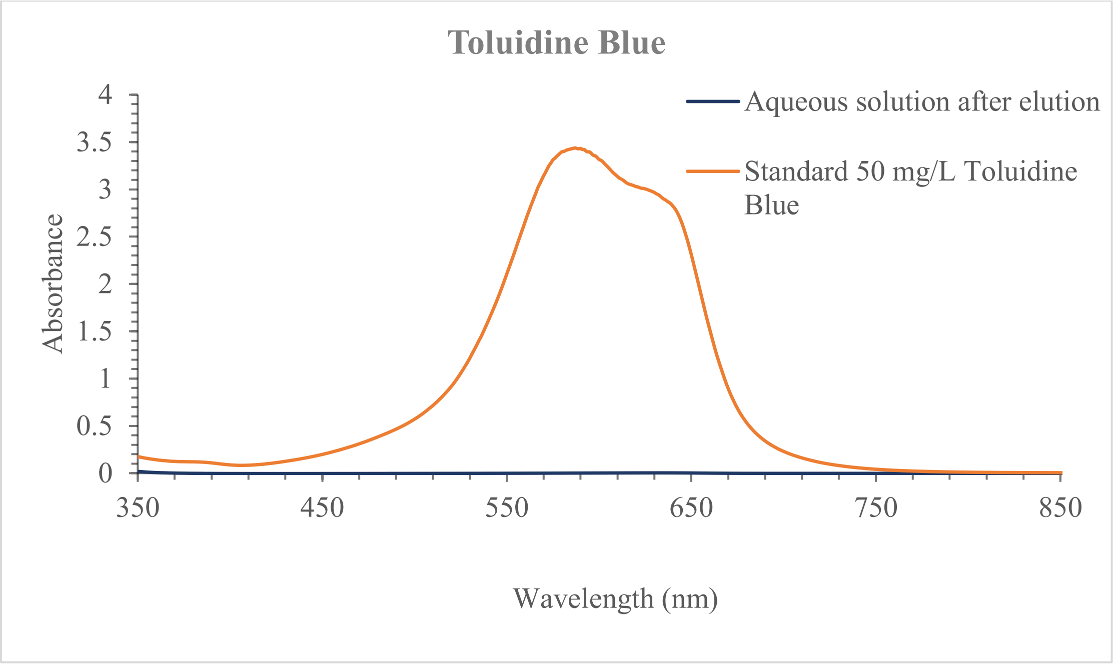

Figure S20. UV-Vis spectra of recovered aqueous layer and the initial toluidine blue solution.

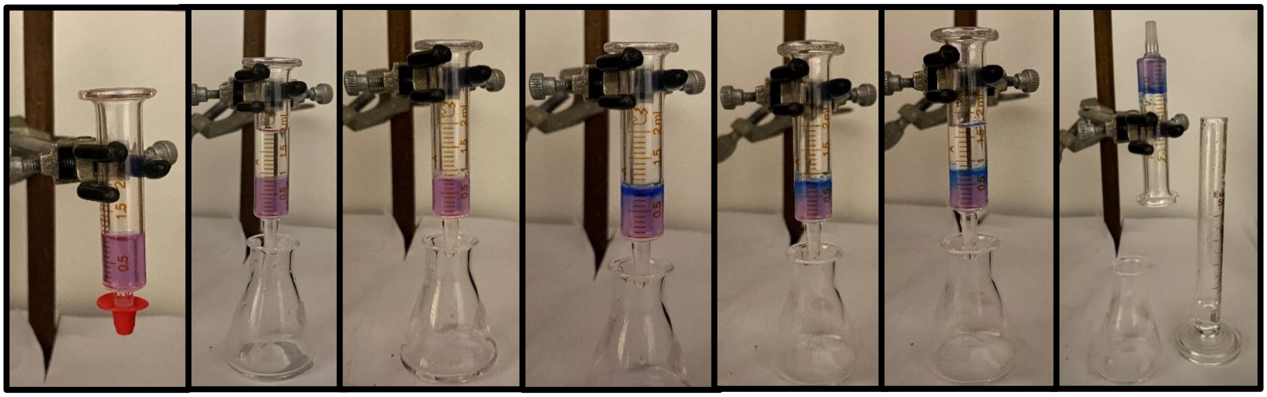

a b c d e f g

Figure S21. (a) The gel column formed by **7C** in 1.0 mL DMSO: H_2O (v/v 1:2) at 4.0 mg/mL, (b) after addition of 1 mL DI water, (c) the gel column after water elution, (d) after loading with 0.1 mL Toluidine blue dye, (e) after 0.1 mL toluidine blue dye passed, (f) 1.0 mL water was added to the gel column in (e), (g) inverted gel column with collected aqueous solution.

2.3 Rhodamine B dye absorption using the gel formed by compound **7O**

The same procedure in section 2.2.1 was used here. A gel was prepared similarly 1.0 mL at 4.0 mg/mL. After the gel was prepared in a syringe column, 1.0 mL water was used to flush some of the DMSO from gel column, however, the gel column shrank to approximately 0.5 mL during the elution. This gel was continued with the dye removal experiment. Rhodamine B dye solution (0.1 mL, 20 mg/L) was added on top of the gel and left settling for 30 min and the solution was eluted from the gel column, after the dye finished eluting, 1.0 mL water was added and eluted again, total 1.2 mL liquid was collected. This was diluted to 2.2 mL and observed on the UV-Vis spectrometer. The absorbance at λ_{max} (554 nm) of Rhodamine B was measured using a calibration curve, about 99.5% of the dye from the initial solution.

UV absorption λ_{max} (554 nm) = 0.002833

From calibration curve: $y = 0.2568x + 0.0024$

$$0.002833 = 0.2568X + 0.0024 \rightarrow x = (0.002833 - 0.0024)/0.2568 = 0.000433\ mg/L$$

Amount of RB after column: 0.000433 mg/L × (1 L/1000 mL) × 2.2 mL = 0.0000095 mg

Original amount of RB added to column: 20 mg/L × (1 L/1000 mL) × 0.1 mL=0.002 mg

Amount of RB removed from the solution: 0.0019 mg, 99.5%

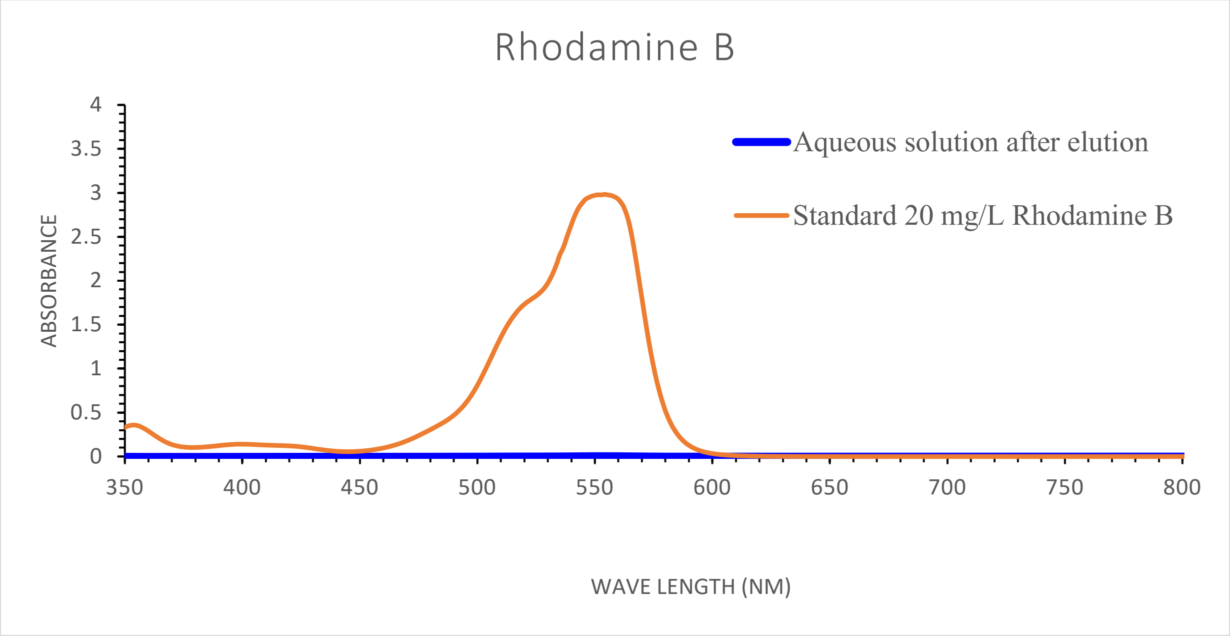

Figure S22. Overlay of UV-Vis spectra of recovered aqueous layer from the study carried out using and the initial Rhodamine B solution.

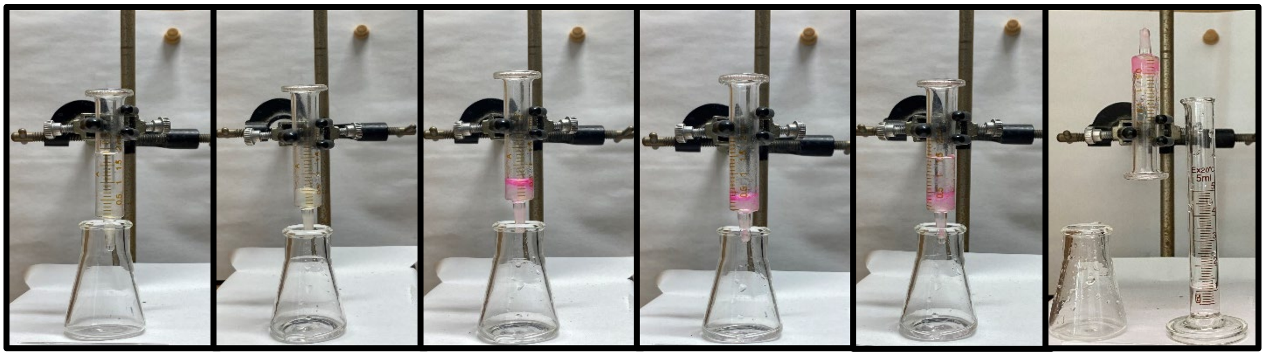

a b c d e f

Figure 23. (a) Gel column with 1 mL DI water; (b) Gel column after flushing with DI water; (c) Gel column after loading with 0.1 mL Rhodamine B dye; (d) Gel column after passing the dye (e) 1 mL of DI water was added to the column; (f) Inverted gel column after the completion of the experiment with aqueous solution.

IV. FT-IR spectra

Compound **7** (2.0 mg) was dissolved in 1.2 mL DMSO: H_2O (v/v 1:2) mixture. The mixture was heated to form a gel. A small piece of gel was placed on the disc of Bruker Alpha FTIR spectrometer and analyzed using OPUS software.

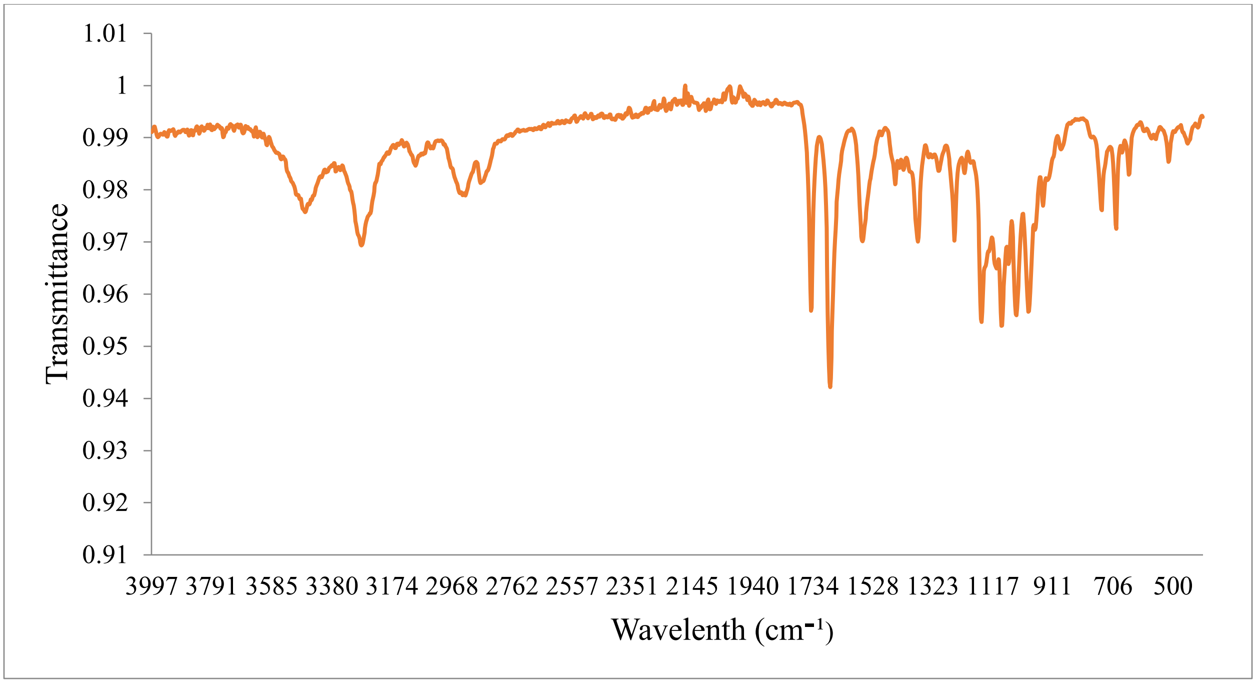

Figure S24. FT-IR spectrum of compound **5**.

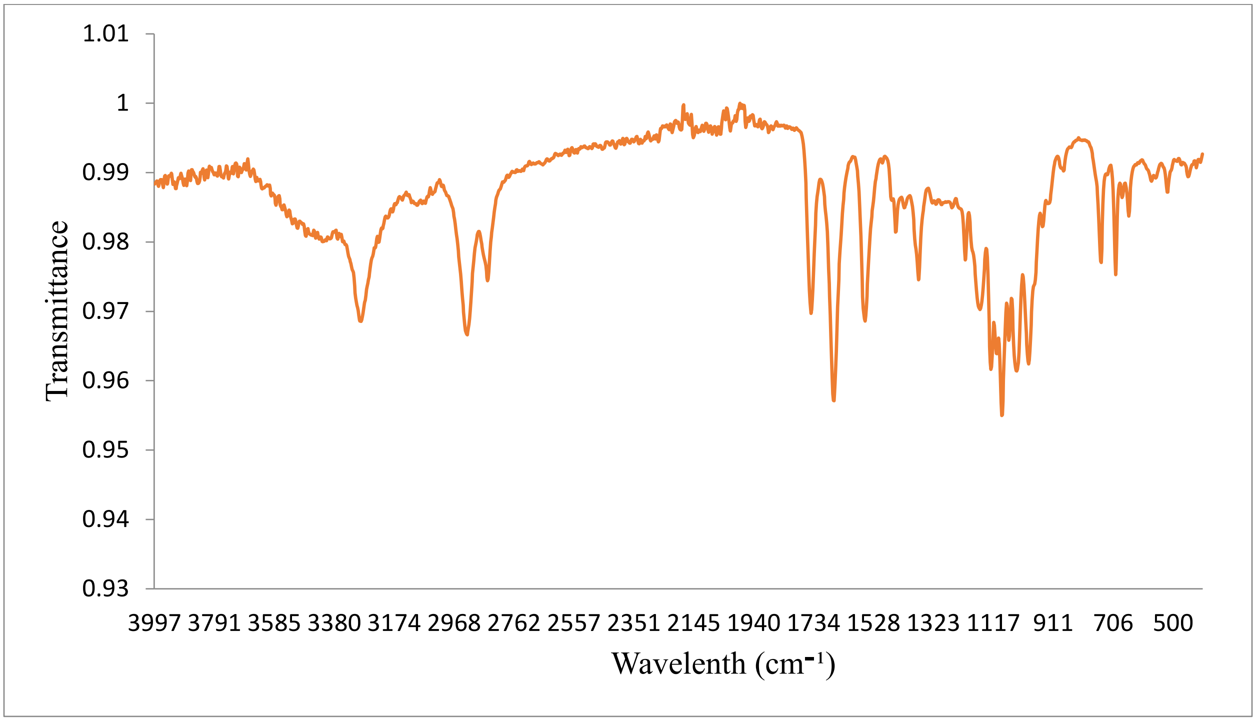

Figure S25. FT-IR spectrum of compound **6**.

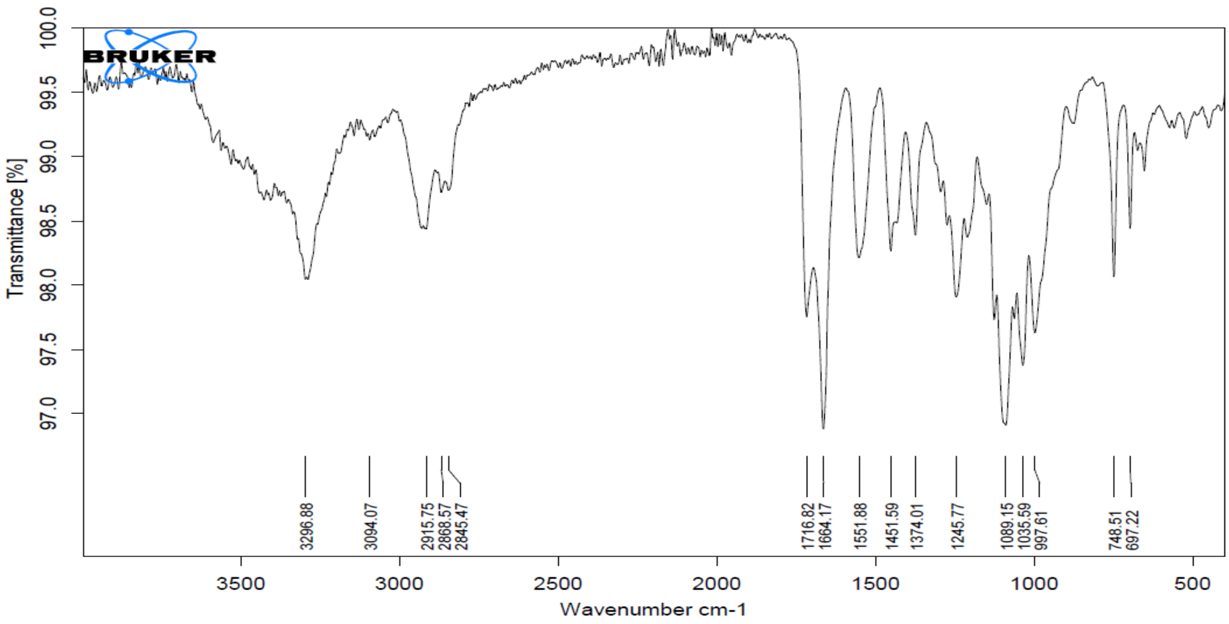

Figure S26. FT-IR spectrum of compound **7**.

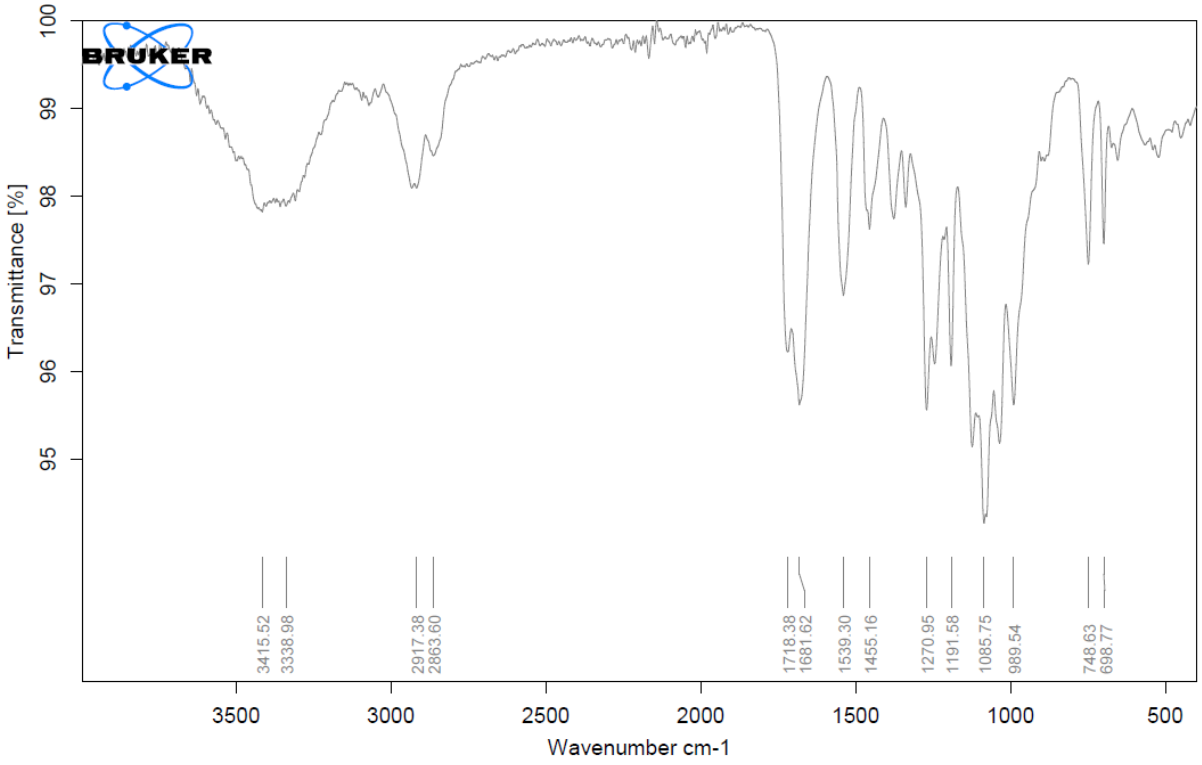

Figure S27. FT-IR spectrum of compound **8**.

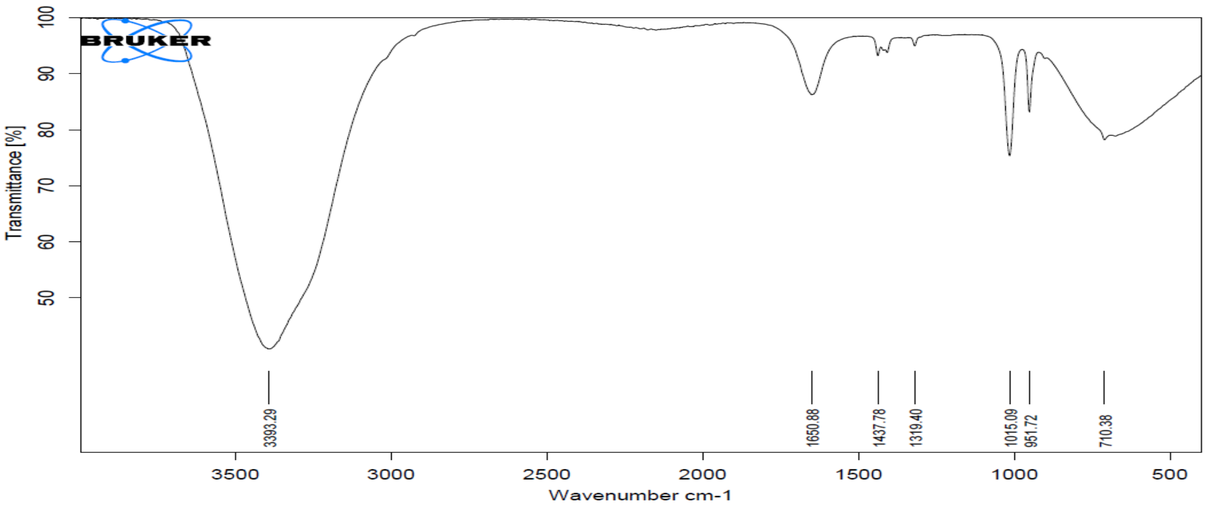

Figure S28. FT-IR spectrum of the gel formed by compound **7** in DMSO: H_2O (v/v 1:2).

V. Images of ODU logo

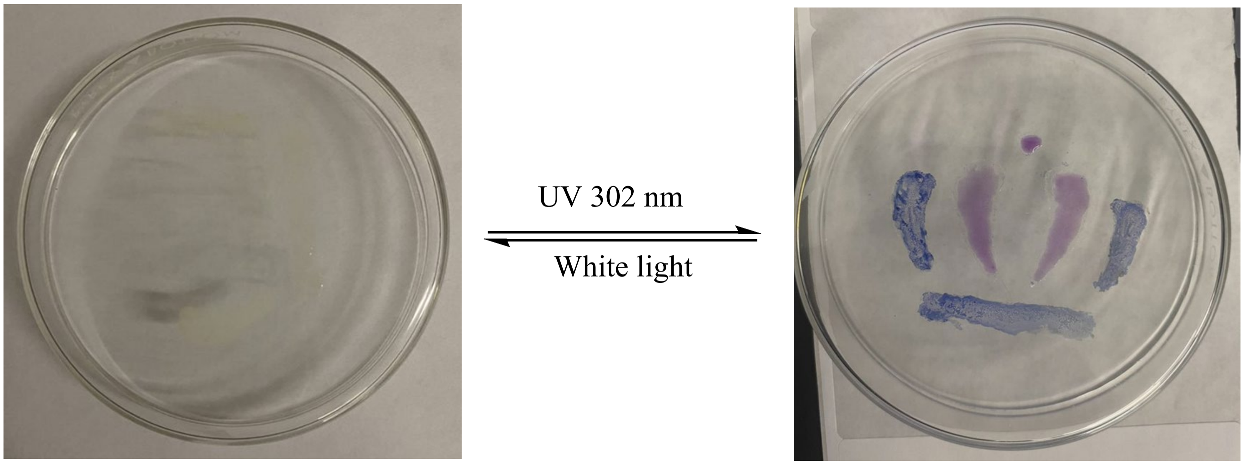

Figure S29. Old Dominion University logo formed by gel of compound **7** and **8** before (left) and after (right) exposure to UV.
